# Supplementary material for: Allosteric modulation of GPCR-induced β-arrestin trafficking and signaling by a synthetic intrabody
Source: Nat Commun. 2022 Aug 8;13:4634. doi: 10.1038/s41467-022-32386-x (PMC9360436; doi:10.1038/s41467-022-32386-x)
Supplement: Supplementary file 1 — Supplementary Information [file 41467_2022_32386_MOESM1_ESM.pdf]

Supplementary information

Allosteric modulation of GPCR-induced β-arrestin trafficking and signaling by a synthetic intrabody

Mithu Baidya[1#], Madhu Chaturvedi[1#], Hemlata Dwivedi-Agnihotri[1#], Ashutosh Ranjan[1], Dominic Devost[2], Yoon Namkung[3], Tomasz Maciej Stepniewski[4,5], Shubhi Pandey[1], Minakshi Baruah[1], Bhanupriya Panigrahi[1], Parishmita Sarma[1], Manish K. Yadav[1], Jagannath Maharana[1], Ramanuj Banerjee[1], Kouki Kawakami[6], Asuka Inoue[6], Jana Selent[4], Stéphane A. Laporte[2,3], Terence E. Hébert[2] and Arun K. Shukla[1*]

- Supplementary Figure 1
- Supplementary Figure 2
- Supplementary Figure 3
- Supplementary Figure 4
- Supplementary Figure 5
- Supplementary Figure 6

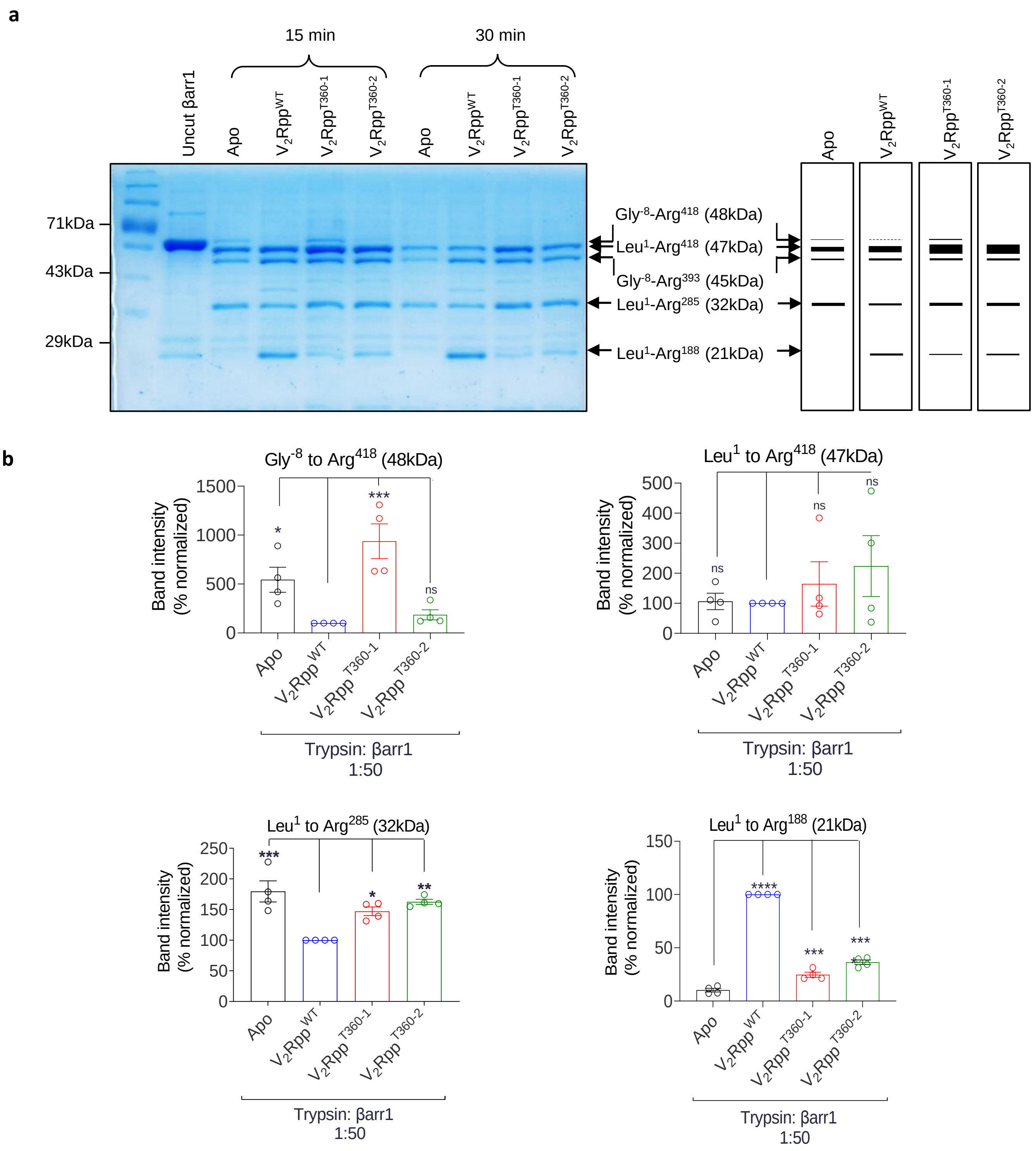
a
15 min
30 min
Uncut βarr1
Apo
V2RppWT
V2RppT360-1
V2RppT360-2
Apo
V2RppWT
V2RppT360-1
V2RppT360-2
71kDa
43kDa
29kDa
Gly-8-Arg418 (48kDa)
Leu1-Arg418 (47kDa)
Gly-8-Arg393 (45kDa)
Leu1-Arg285 (32kDa)
Leu1-Arg188 (21kDa)
Apo
V2RppWT
V2RppT360-1
V2RppT360-2
b
Gly-8 to Arg418 (48kDa)
Band intensity (% normalized)
1500
1000
500
0
*
***
ns
Apo
V2RppWT
V2RppT360-1
V2RppT360-2
Trypsin: βarr1 1:50
Leu1 to Arg418 (47kDa)
Band intensity (% normalized)
500
400
300
200
100
0
ns
ns
ns
Apo
V2RppWT
V2RppT360-1
V2RppT360-2
Trypsin: βarr1 1:50
Leu1 to Arg285 (32kDa)
Band intensity (% normalized)
250
200
150
100
50
0
***
*
**
Apo
V2RppWT
V2RppT360-1
V2RppT360-2
Trypsin: βarr1 1:50
Leu1 to Arg188 (21kDa)
Band intensity (% normalized)
150
100
50
0
****
***
***
Apo
V2RppWT
V2RppT360-1
V2RppT360-2
Trypsin: βarr1 1:50

Supplementary Figure 1. Limited trypsin proteolysis of βarr1: (a) The trypsin digestion pattern of V_2Rpp^{WT}, V_2Rpp^{T360-1} or V_2Rpp^{T360-2} bound βarr1 is shown at indicated time points. βarr1 activated with 50-fold molar excess of different phosphopeptides was subjected to limited trypsin proteolysis at a trypsin: βarr1 ratio of 1:50. The proteolysis reaction was quenched with SDS buffer after 15 and 30min, and the digested fragments were separated by SDS-PAGE. There are evident differences in the fragments (Leu^1-Arg^{418} and Leu^1-Arg^{188}) resulting from digestion of ligand-free and ligand bound (V_2Rpp^{WT}, V_2R^{T360-1} or V_2Rpp^{T360-2}) βarr1. **(b).** Densitometry based quantification (mean±SEM) of the band intensities for different tryptic fragments generated from βarr1 at 15min time-point. Data from four independent experiments, normalized with respect to V_2R^{WT} condition, and analyzed using One-way ANOVA (mean±SEM; Dunnett's multiple comparisons test) is presented here. The exact p values are as follows: Gly^{-8} to Arg^{418} (48kDa) band- Apo (p= 0.041), V_2R^{T360-1} (p= 0.0006), V_2R^{T360-2} (p=0.909); Leu^1 to Arg^{418} (47kDa) band- Apo(p=0.9997), V_2R^{T360-1} (p= 0.8193), V_2R^{T360-2} (p=0.4146); $Leul^1$ to Arg^{285} (32kDa) band- Apo(p=0.0002), V_2R^{T360-1} (p= 0.0113), V_2R^{T360-2} (p=0.0015); $Leul^1$ to Arg^{188} (21kDa) band- Apo,V_2R^{T360-1}, V_2R^{T360-2} (p<0.0001). Source data are provided as a Source Data file (*p<0.05, **p<0.01, ***p<0.001, **** p<0.0001).

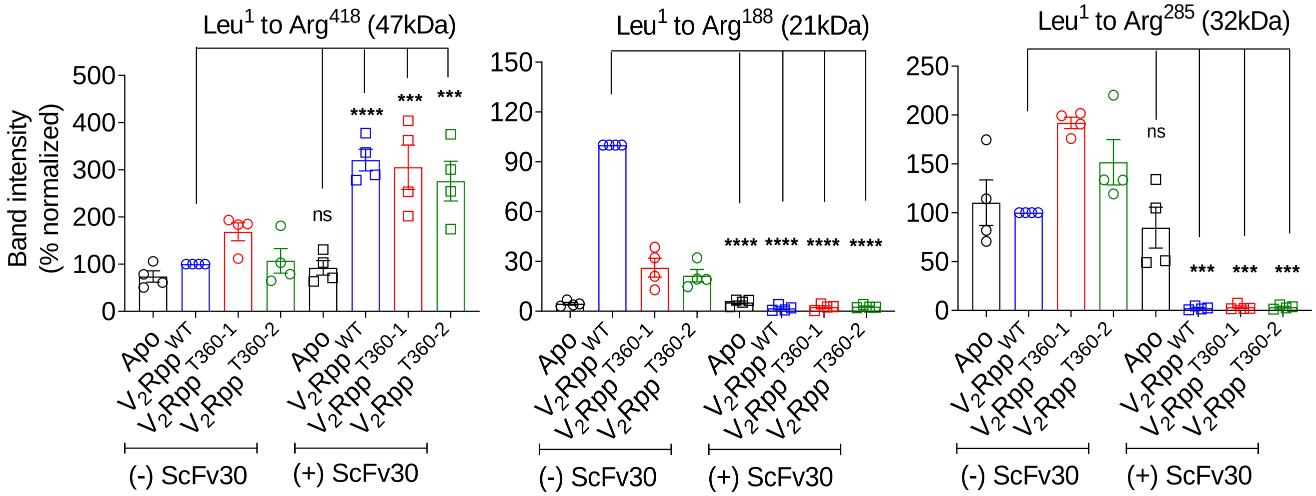

Supplementary Figure 2. Densitometry based quantification (mean±SEM) of trypsin fragments corresponding to 47kDa (Leu^1-Arg^{418}), 32kDa (Leu^1-Arg^{285}) and 21kDa (Leu^1-Arg^{188}) generated in presence and absence of ScFv30 is shown (based on data presented in Figure 2E). Data from four independent experiments, normalized with respect to V_2R^{WT}- without ScFv30 condition, and analyzed using One-way ANOVA (mean±SEM; Dunnett's multiple comparisons test) is presented here. The exact p values are as follows: Leu^1 to Arg^{418} (47kDa) band- Apo (p=0.9997), V_2R^{WT} (p <0.0001), V_2R^{T360-1} (p =0.0001), V_2R^{T360-2} (p =0.0008); Leul^1 to Arg^{188} (21kDa) band- Apo, V_2R^{WT} ,V_2R^{T360-1}, V_2R^{T360-2} (p<0.0001); Leul^1 to Arg^{285} (32kDa) band- Apo (p=0.9503), V_2R^{WT} (p=0.0003), V_2R^{T360-1} (p =0.0003), V_2R^{T360-2} (p =0.0004) . Source data are provided as a Source Data file. (***p<0.001, **** p<0.0001)

4JQI			7DFA		
Residue	Interacting partner residue	Distance (Å)	Residue	Interacting partner residue	Distance (Å)
188 ARG N	197 LEU C	4.00	188 ARG CG	190 PHE CE1	3.68
188 ARG N	197 LEU O	2.90	188 ARG CG	190 PHE CZ	3.63
188 ARG CA	197 LEU O	3.84	188 ARG NE	190 PHE CE1	3.97
188 ARG C	197 LEU N	3.90	188 ARG NH2	341 SER CA	3.95
188 ARG O	196 PRO CA	3.42	188 ARG NH2	340 SER O	3.17
188 ARG O	196 PRO C	3.58			
188 ARG O	197 LEU N	2.77			
188 ARG O	197 LEU CA	3.73			
188 ARG O	197 LEU O	3.76			
188 ARG O	197 LEU CB	3.81			
188 ARG O	190 PHE N	3.99			
188 ARG O	196 PRO CB	3.72			
188 ARG CB	197 LEU O	3.94			
188 ARG CB	343 VAL CG2	3.57			
188 ARG CG	190 PHE CZ	3.57			
188 ARG CG	343 VAL CG2	3.83			
188 ARG CG	190 PHE CE1	3.52			
188 ARG NH1	341 SER C	3.84			
188 ARG NH1	341 SER O	3.84			
188 ARG NH1	342 ASP C	3.89			
188 ARG NH1	342 ASP O	2.78			
188 ARG NH2	340 SER O	3.07			
285 ARG N	283 GLU C	3.43	285 ARG CB	130 GLN O	3.98
285 ARG N	283 GLU O	3.13	285 ARG CG	130 GLN O	3.55
285 ARG CA	130 GLN O	3.45	285 ARG CG	132 GLY N	3.66
285 ARG C	130 GLN O	3.58	285 ARG CD	132 GLY CA	3.85
285 ARG C	287 LEU N	3.27	285 ARG CD	132 GLY N	3.55
285 ARG O	336 GLY CA	3.91	285 ARG CD	135 ASP OD2	3.31
285 ARG O	287 LEU N	3.55	285 ARG NE	135 ASP CG	3.70
285 ARG O	335 LEU C	3.80	285 ARG NE	135 ASP OD1	3.53
285 ARG O	335 LEU O	3.47	285 ARG NE	135 ASP OD2	3.04
285 ARG O	338 LEU CG	3.51	285 ARG CZ	333 GLY O	3.58
285 ARG O	338 LEU CD1	3.50	285 ARG NH1	336 GLY CA	3.58
285 ARG CB	130 GLN O	3.89	285 ARG NH1	336 GLY N	3.89
285 ARG CG	130 GLN O	3.26	285 ARG NH1	333 GLY O	3.47
285 ARG CG	131 PRO C	3.86	285 ARG NH2	135 ASP OD1	3.82
285 ARG CG	132 GLY N	3.61	285 ARG NH2	333 GLY O	3.04
285 ARG CD	336 GLY CA	3.80			
285 ARG CD	333 GLY O	3.29			
285 ARG CD	336 GLY N	3.88			
285 ARG NE	333 GLY O	3.41			
285 ARG CZ	333 GLY O	3.64			
285 ARG NH1	135 ASP CG	3.99			
285 ARG NH1	135 ASP OD1	3.85			
285 ARG NH1	135 ASP OD2	3.33			
285 ARG NH1	333 GLY O	3.72			

Supplementary Figure 3. Inter residue contacts (within 4Å) corresponding to residues Arg^{188} and Arg^{285} of 4JQI and 7DFA; calculated separately using CONTACT/ACT program within the CCP4 suite.

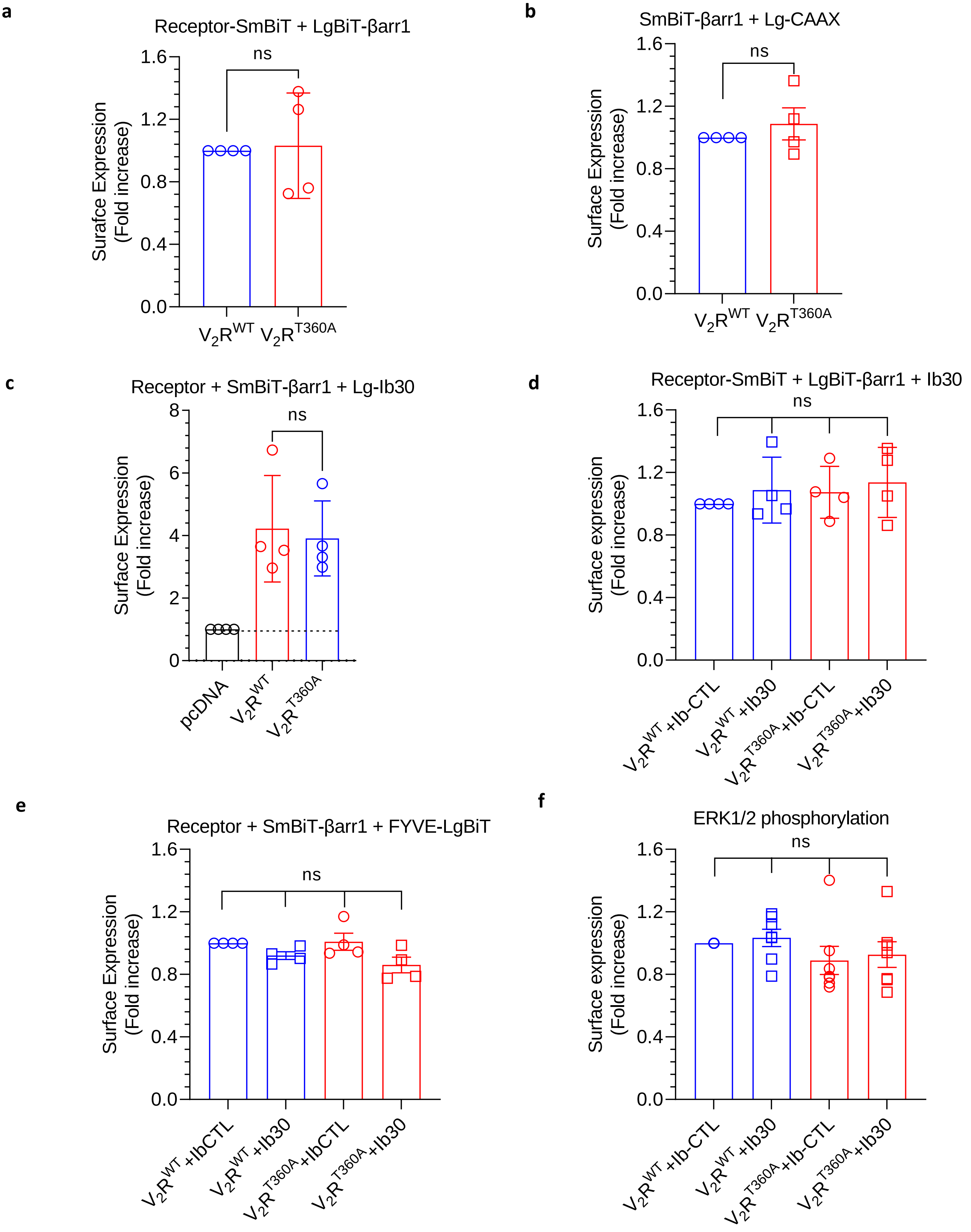
a
Receptor-SmBiT + LgBiT-βarr1
Surafce Expression
(Fold increase)
ns
V2RWT V2RT360A
b
SmBiT-βarr1 + Lg-CAAX
Surface Expression
(Fold increase)
ns
V2RWT V2RT360A
c
Receptor + SmBiT-βarr1 + Lg-Ib30
Surface Expression
(Fold increase)
ns
pcDNA
V2RWT
V2RT360A
d
Receptor-SmBiT + LgBiT-βarr1 + Ib30
Surface expression
(Fold increase)
ns
V2RWT +Ib-CTL
V2RWT +Ib30
V2RT360A+Ib-CTL
V2RT360A+Ib30
e
Receptor + SmBiT-βarr1 + FYVE-LgBiT
Surface Expression
(Fold increase)
ns
V2RWT +IbCTL
V2RWT +Ib30
V2RT360A+IbCTL
V2RT360A+Ib30
f
ERK1/2 phosphorylation
Surface expression
(Fold increase)
ns
V2RWT +Ib-CTL
V2RWT +Ib30
V2RT360A+Ib-CTL
V2RT360A+Ib30

Supplementary Figure 4. Surface expression of V_2R constructs in different assays. (a-e) Surface expression of V_2R constructs in the NanoBiT assay as measured using whole cell ELISA, normalized with respect to V_2R^{WT} (treated as 100%), and analyzed using paired t-test (mean±SEM from four independent experiments, ns, non-significant). **(f).** Surface expression of V_2R constructs in ERK1/2 phosphorylation assays as measured using whole cell ELISA, normalized with respect to vector-transfected cells (treated as 1), and analyzed using One-way ANOVA (mean±SEM from seven independent experiments, Sidak's multiple comparisons test ns, non-significant). Source data are provided as a Source Data file**.**

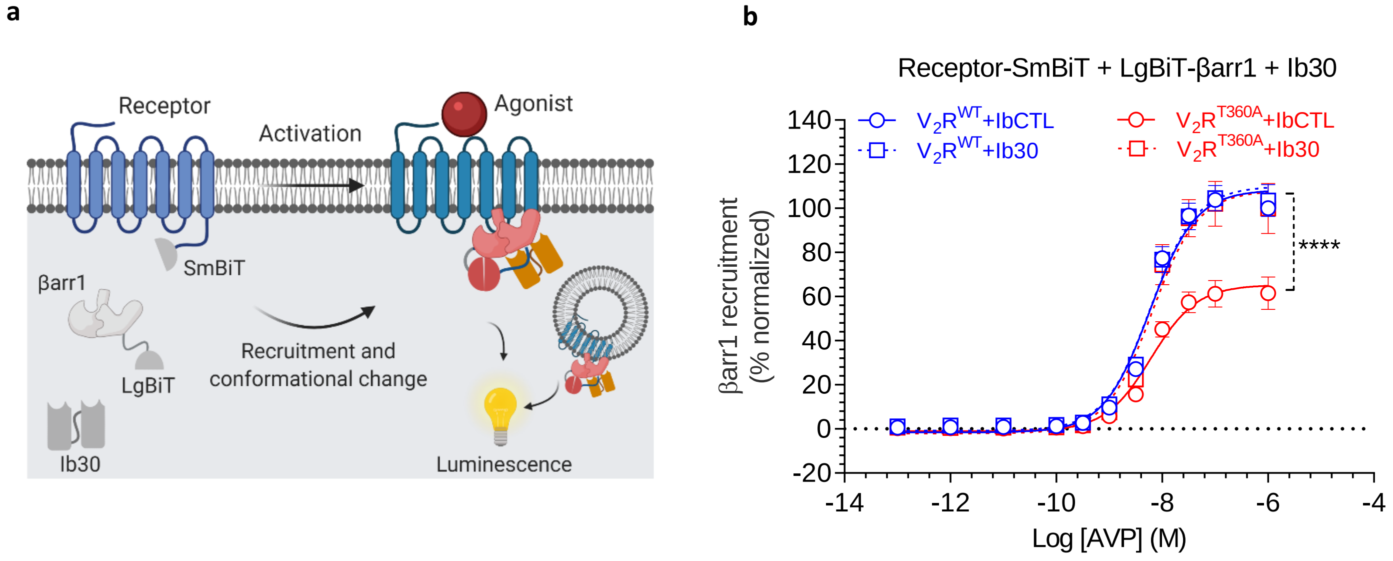

Supplementary Figure 5. (a) Schematic representation of NanoBiT-based βarr1 recruitment assay (created with BioRender.com). **(b)** HEK-293 cells expressing the indicated receptor and βarr1 constructs together with Ib-CTL or Ib30 were stimulated with varying doses of AVP for 30min followed by the measurement of luminescence (mean±SEM from four independent experiments; normalized with luminescence signal for V_2R^{WT}at maximal ligand dose as 100%) (Two-way ANOVA, Tukey's multiple comparisons test; ****p<0.0001). Source data are provided as a Source Data file**.**

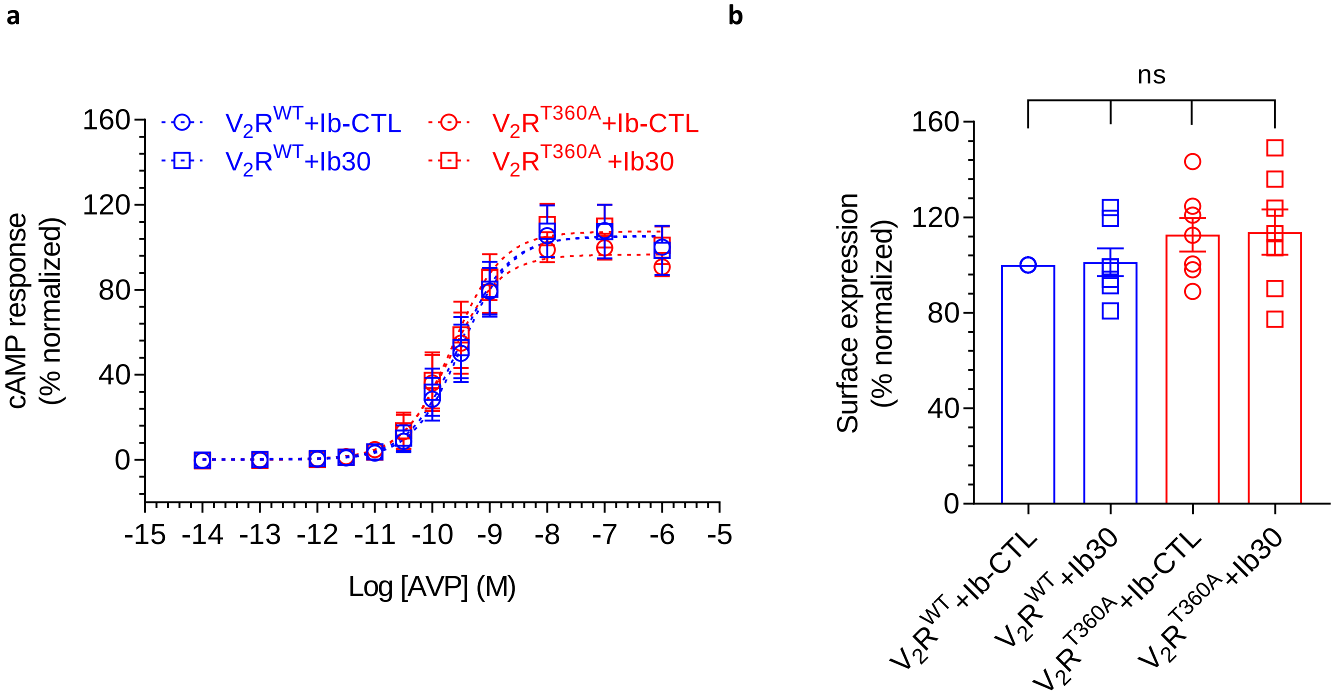

Supplementary Figure 6. Effect of Intrabody30 (Ib30) on agonist-induced cAMP response. (a) Ib30 does not affect agonist-induced cAMP response for the V_2R constructs as assessed using GloSensor assay. HEK-293 cells expressing the indicated constructs were stimulated with AVP followed by measurement of cAMP using luminescence-based readout. Data (mean±SEM) from seven independent experiments are presented here. **(b).** Surface expression of V_2R constructs in GloSensor assay as measured using whole cell ELISA (mean ±SEM, from seven independent experiments, One-way ANOVA, Dunnett's multiple comparison test). Source data are provided as a Source Data file**.**
