# Supplementary material for: Allosteric modulation of GPCR-induced β-arrestin trafficking and signaling by a synthetic intrabody
Source: Nat Commun. 2022 Aug 8;13:4634. doi: 10.1038/s41467-022-32386-x (PMC9360436; doi:10.1038/s41467-022-32386-x)
Supplement: Supplementary file 3 — Reporting Summary [file 41467_2022_32386_MOESM3_ESM.pdf]

nature portfolio

Corresponding author(s): Arun K. Shukla

Last updated by author(s): 11-07-2022

Reporting Summary

Nature Portfolio wishes to improve the reproducibility of the work that we publish. This form provides structure for consistency and transparency in reporting. For further information on Nature Portfolio policies, see our Editorial Policies and the Editorial Policy Checklist.

Statistics

For all statistical analyses, confirm that the following items are present in the figure legend, table legend, main text, or Methods section.

n/a	Confirmed	
☐	☒	The exact sample size (*n*) for each experimental group/condition, given as a discrete number and unit of measurement
☐	☒	A statement on whether measurements were taken from distinct samples or whether the same sample was measured repeatedly
☐	☒	The statistical test(s) used AND whether they are one- or two-sided<br>*Only common tests should be described solely by name; describe more complex techniques in the Methods section.*
☒	☐	A description of all covariates tested
☐	☒	A description of any assumptions or corrections, such as tests of normality and adjustment for multiple comparisons
☐	☒	A full description of the statistical parameters including central tendency (e.g. means) or other basic estimates (e.g. regression coefficient) AND variation (e.g. standard deviation) or associated estimates of uncertainty (e.g. confidence intervals)
☐	☒	For null hypothesis testing, the test statistic (e.g. *F*, *t*, *r*) with confidence intervals, effect sizes, degrees of freedom and *P* value noted<br>*Give P values as exact values whenever suitable.*
☒	☐	For Bayesian analysis, information on the choice of priors and Markov chain Monte Carlo settings
☒	☐	For hierarchical and complex designs, identification of the appropriate level for tests and full reporting of outcomes
☒	☐	Estimates of effect sizes (e.g. Cohen's *d*, Pearson's *r*), indicating how they were calculated

Our web collection on statistics for biologists contains articles on many of the points above.

Software and code

Policy information about availability of computer code

Data collection	MOE package (v2015.10)<br>Charmm36M forcefield (v36M)<br>ACEMD3 engine (v3.5.0)
Data analysis	ImageJ, NIH (v1.80_172)<br>GraphPad Prism (v9.3)<br>GraphPad Prism (v6)<br>Zen Black 2012 SP1, Zeiss (v8.1.0484)<br>Image lab, BioRad (v6.1)<br>UCSF Chimera X (v1.2)<br>CONTACT/ACT program within the CCP4 suite (v.7.1.018)

For manuscripts utilizing custom algorithms or software that are central to the research but not yet described in published literature, software must be made available to editors and reviewers. We strongly encourage code deposition in a community repository (e.g. GitHub). See the Nature Portfolio guidelines for submitting code & software for further information.

Data

Policy information about availability of data

All manuscripts must include a data availability statement. This statement should provide the following information, where applicable:

- Accession codes, unique identifiers, or web links for publicly available datasets
- A description of any restrictions on data availability
- For clinical datasets or third party data, please ensure that the statement adheres to our policy

The original raw data for gels, immunoblots and confocal micrographs have been deposited in Mendeley Data (doi: 10.17632/8wmkcw8ht7.1). This paper does not report any original code. The coordinates for V2RppWT-βarr1 and V2RppT360-1-βarr1 crystal structures used in this study are available in PDB with ID 4JQI and 7DFA, respectively. Any additional information required to reanalyze the data reported in this paper is available from the corresponding author upon reasonable request. Source data are provided with this paper. Original data pertaining to MD simulation are deposited in GPCRmd (https://submission.gpcrmd.org/dynadb/publications/1486/).

Human research participants

Policy information about studies involving human research participants and Sex and Gender in Research.

Reporting on sex and gender	NA
Population characteristics	NA
Recruitment	NA
Ethics oversight	NA

Note that full information on the approval of the study protocol must also be provided in the manuscript.

Field-specific reporting

Please select the one below that is the best fit for your research. If you are not sure, read the appropriate sections before making your selection.

☒ Life sciences ☐ Behavioural & social sciences ☐ Ecological, evolutionary & environmental sciences

For a reference copy of the document with all sections, see nature.com/documents/nr-reporting-summary-flat.pdf

Life sciences study design

All studies must disclose on these points even when the disclosure is negative.

Sample size	As appropriate for different experimental assays, we carried out at least three independent biological replicates for each experiment (see corresponding figure legends in main and supplementary figures for exact number of replicates). Minimal sample sizes were chosen based on the sample variability and heterogeneity, and they were not predetermined using any statistical method. The sample sizes selected for this study were sufficient to define the differences between groups.
Data exclusions	No data exclusion were performed.
Replication	Multiple replicates of the experiments were performed including both technical and biological replicates. The details of the number of biological replicates are outlined in each individual figure legend. All replications were successful.
Randomization	Since this is not an animal or clinical case/control study, randomization was neither attempted nor needed. Due to the quantitative nature of biological research studies, no covariates were considered and the independent covariates tested were sufficient for the functional interpretation in this study.
Blinding	Blinding was done for cell counting from confocal micrographs to avoid any discrepancies and biases that may arise from manual counting. Three different individuals counted the same images that were blinded. The final tally was cross-checked at the end (Figure 5F).<br>For other experiments blinding was not necessary or possible for cell-based functional assays. Because in most cases, data were collected and analyzed by the individual investigators.

Reporting for specific materials, systems and methods

We require information from authors about some types of materials, experimental systems and methods used in many studies. Here, indicate whether each material, system or method listed is relevant to your study. If you are not sure if a list item applies to your research, read the appropriate section before selecting a response.

Materials & experimental systems

n/a	Involved in the study
☐	☒ Antibodies
☐	☒ Eukaryotic cell lines
☒	☐ Palaeontology and archaeology
☒	☐ Animals and other organisms
☒	☐ Clinical data
☒	☐ Dual use research of concern

Methods

n/a	Involved in the study
☒	☐ ChIP-seq
☒	☐ Flow cytometry
☒	☐ MRI-based neuroimaging

Antibodies

Antibodies used

1. Phospho-p44/42 MAPK (Erk1/2) (Thr202/Tyr204) Antibody, Supplier: Cell Signaling Technology (CST), #Cat. no. 9101, Lot no. 30
2. p44/42 MAPK (Erk1/2) Antibody, Supplier: Cell Signaling Technology (CST), #Cat. no. 9102, Lot no. 26.
3. Anti-Rabbit IgG (whole molecule)–Peroxidase antibody, Supplier: Sigma-Aldrich, #Cat. no. A9169, Lot no. 0000086280.
4. HA-probe Antibody, Supplier: Santa Cruz Biotechnology, #Cat. no. sc-805, Clone Y-11, Lot no. C0615.
5. Monoclonal Anti-β-Actin antibody, Supplier: Sigma-Aldrich, #Cat. no. A3854, Clone AC-15, Lot no. 089M4850V.
6. β-Arrestin 1/2 (D24H9) Rabbit mAb, Supplier: Cell Signaling Technology (CST), #Cat. no. 4674, Clone D24H9, Lot no. 2.
7. HRP-Protein L, Supplier: GenScript, #Cat. no. M00098, Lot no. 14J000836.
8. Monoclonal ANTI-FLAG® M2-Peroxidase (HRP) antibody. Supplier: Sigma-Aldrich, #Cat. no. A8592, Clone M2, Lot no. SLBV3799.
9. Anti-HA-peroxidase conjugate. Supplier: Sigma-Aldrich, #Cat. no. 12013819001, Clone 3F10, Lot no. 23551600.

Validation

All antibodies used in this study are well characterized and were applied according to data sheet information details.
1. Phospho-p44/42 MAPK (Erk1/2) (Thr202/Tyr204) Antibody: https://www.cellsignal.com/products/primary-antibodies/phospho-p44-42-mapk-erk1-2-thr202-tyr204-antibody/9101
2. p44/42 MAPK (Erk1/2) Antibody: https://www.cellsignal.com/products/primary-antibodies/p44-42-mapk-erk1-2-antibody/9102
3. Anti-Rabbit IgG (whole molecule)–Peroxidase antibody: https://www.sigmaaldrich.com/IN/en/product/sigma/a9169
4. HA-probe Antibody: https://www.scbt.com/p/ha-probe-antibody-y-11?requestFrom=search
5. Monoclonal Anti-β-Actin antibody: https://www.sigmaaldrich.com/IN/en/product/sigma/a3854
6. β-Arrestin 1/2 (D24H9) Rabbit mAb: https://www.cellsignal.com/products/primary-antibodies/b-arrestin-1-2-d24h9-rabbit-mab/4674
7. HRP-Protein L: https://www.genscript.com/molecule/M00098-HRP_Protein_L.html
8. Monoclonal ANTI-FLAG® M2-Peroxidase (HRP) antibody: https://www.sigmaaldrich.com/IN/en/product/sigma/a8592
9. anti-HA-peroxidase conjugate: https://www.sigmaaldrich.com/IN/en/product/roche/12013819001

Eukaryotic cell lines

Policy information about cell lines and Sex and Gender in Research

Cell line source(s)	HEK-293 cells were obtained from (ATCC; cat. no. CRL-3216) and Sf9 cells were bought from (Expression Systems; cat. no. 94-001F)
Authentication	None of the cell lines used were authenticated.
Mycoplasma contamination	Cell line were not tested for Mycoplasma contamination.
Commonly misidentified lines (See ICLAC register)	None
