# Supplementary material for: Allosteric modulation of GPCR-induced β-arrestin trafficking and signaling by a synthetic intrabody
Source: Nat Commun. 2022 Aug 8;13:4634. doi: 10.1038/s41467-022-32386-x (PMC9360436; doi:10.1038/s41467-022-32386-x)

Figure 1c

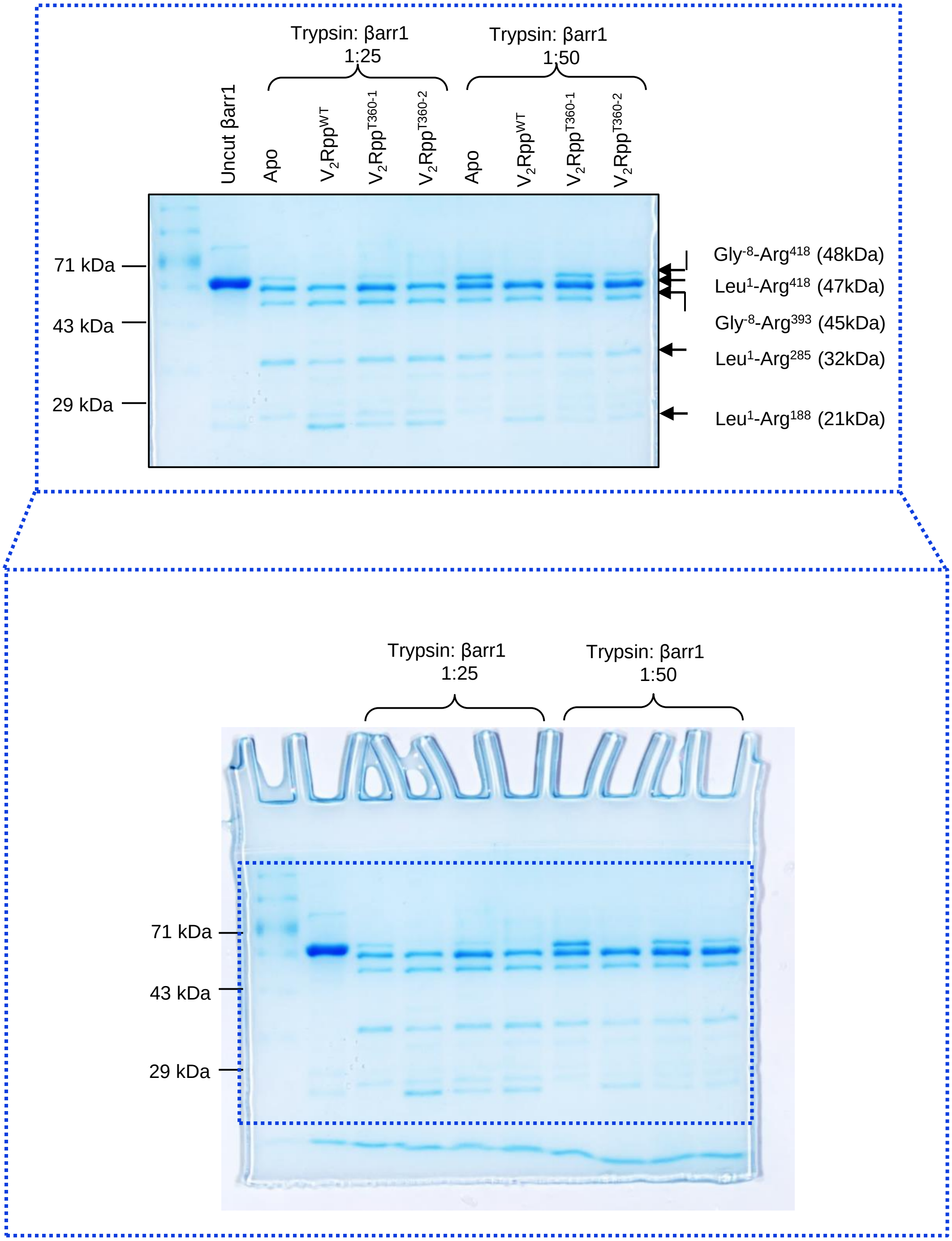

Trypsin: βarr1 1:25
Trypsin: βarr1 1:50
Uncut βarr1
Apo
V2RppWT
V2RppT360-1
V2RppT360-2
Apo
V2RppWT
V2RppT360-1
V2RppT360-2
71 kDa
43 kDa
29 kDa
Gly-8-Arg418 (48kDa)
Leu1-Arg418 (47kDa)
Gly-8-Arg393 (45kDa)
Leu1-Arg285 (32kDa)
Leu1-Arg188 (21kDa)
Trypsin: βarr1 1:25
Trypsin: βarr1 1:50
71 kDa
43 kDa
29 kDa

Figure 2a

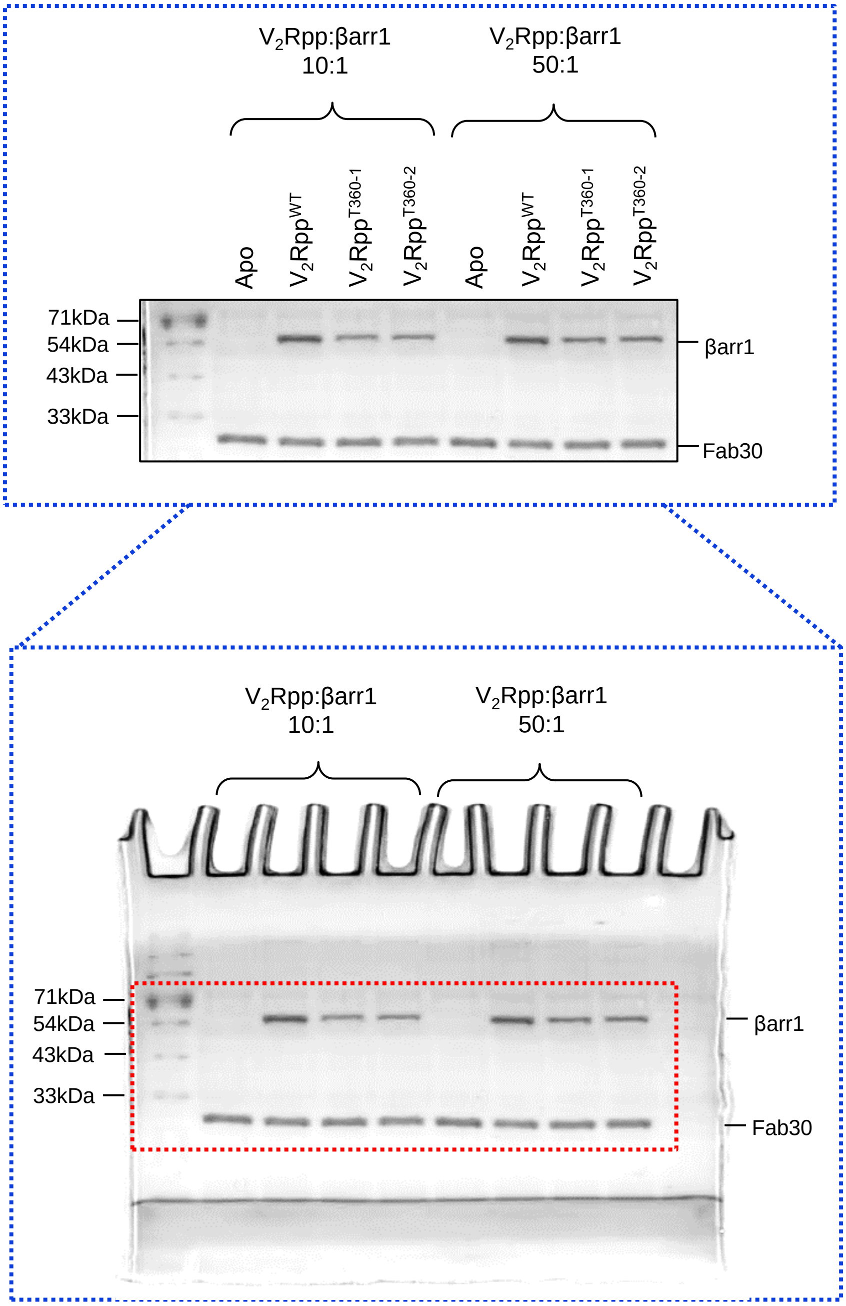
V2Rpp:βarr1
10:1
V2Rpp:βarr1
50:1
Apo
V2RppWT
V2RppT360-1
V2RppT360-2
Apo
V2RppWT
V2RppT360-1
V2RppT360-2
71kDa
54kDa
43kDa
33kDa
βarr1
Fab30
V2Rpp:βarr1
10:1
V2Rpp:βarr1
50:1
71kDa
54kDa
43kDa
33kDa
βarr1
Fab30

Figure 2c

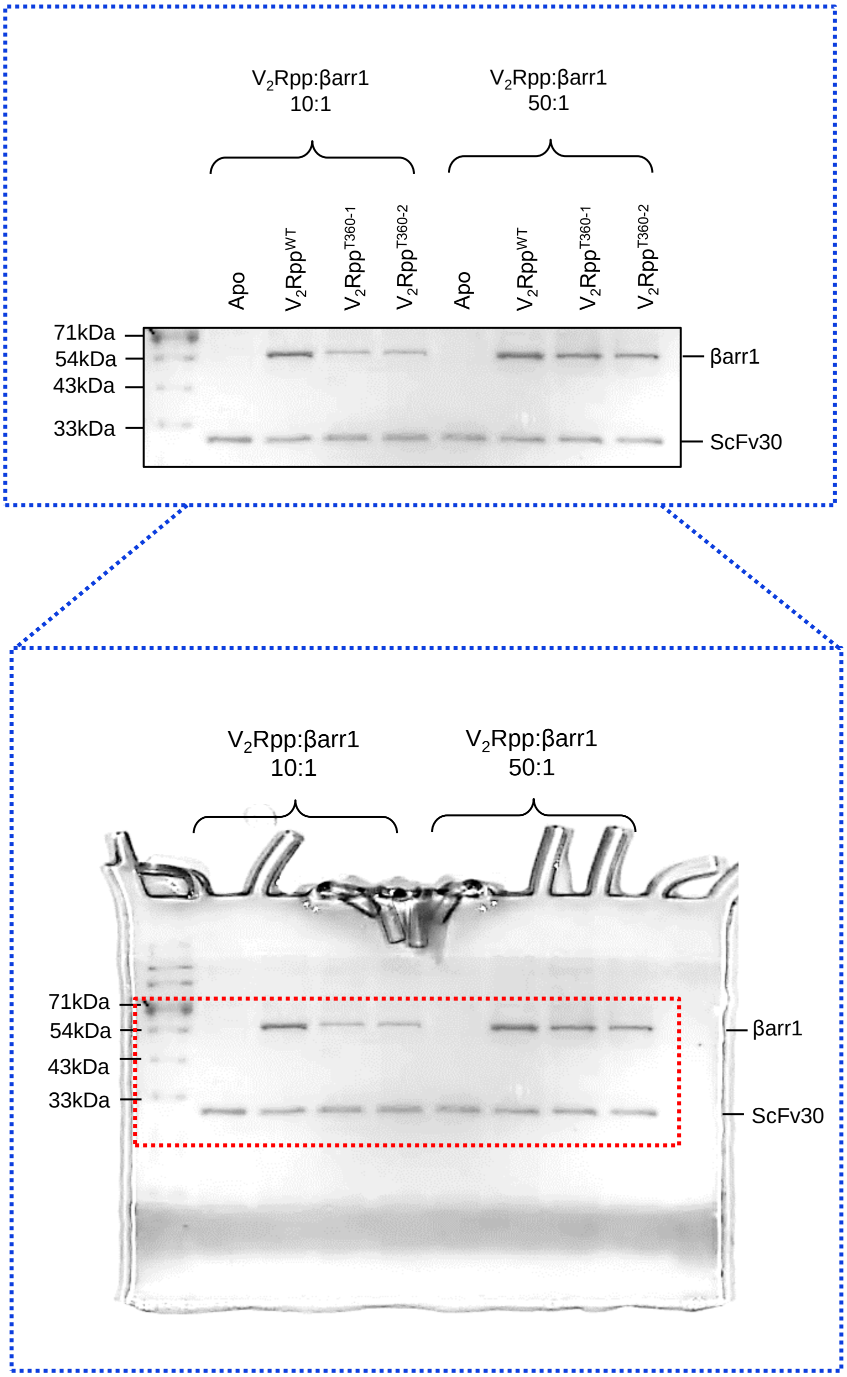
V2Rpp:βarr1
10:1
V2Rpp:βarr1
50:1
Apo
V2RppWT
V2RppT360-1
V2RppT360-2
Apo
V2RppWT
V2RppT360-1
V2RppT360-2
71kDa
54kDa
43kDa
33kDa
βarr1
ScFv30
V2Rpp:βarr1
10:1
V2Rpp:βarr1
50:1
71kDa
54kDa
43kDa
33kDa
βarr1
ScFv30

Figure 2e

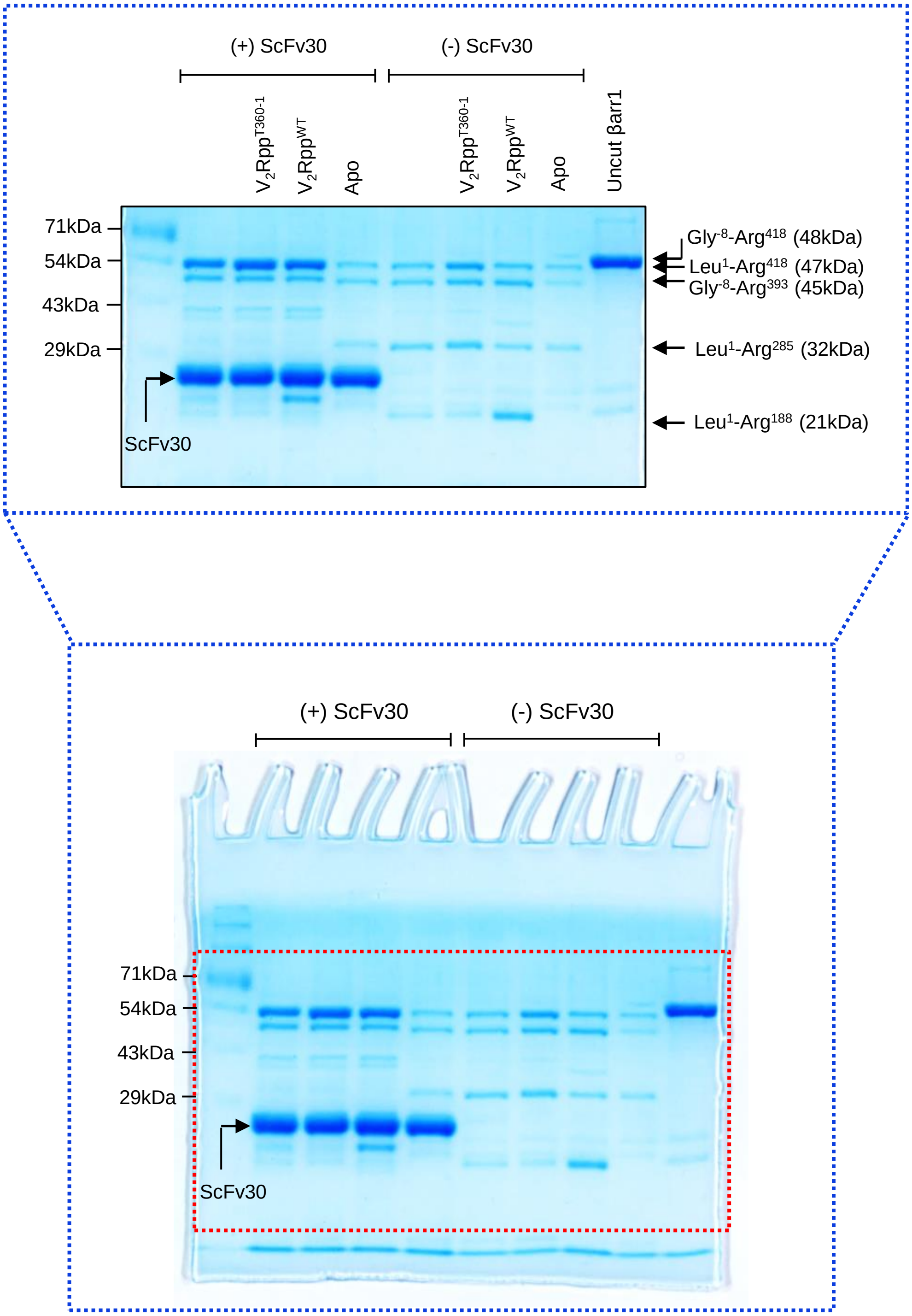

(+) ScFv30
(-) ScFv30
V_2Rpp^{T360-1}
V_2Rpp^{WT}
Apo
V_2Rpp^{T360-1}
V_2Rpp^{WT}
Apo
Uncut βarr1
71kDa
54kDa
43kDa
29kDa
ScFv30
Gly^{-8}-Arg^{418} (48kDa)
Leu^{1}-Arg^{418} (47kDa)
Gly^{-8}-Arg^{393} (45kDa)
Leu^{1}-Arg^{285} (32kDa)
Leu^{1}-Arg^{188} (21kDa)
(+) ScFv30
(-) ScFv30
71kDa
54kDa
43kDa
29kDa
ScFv30

Figure 5c

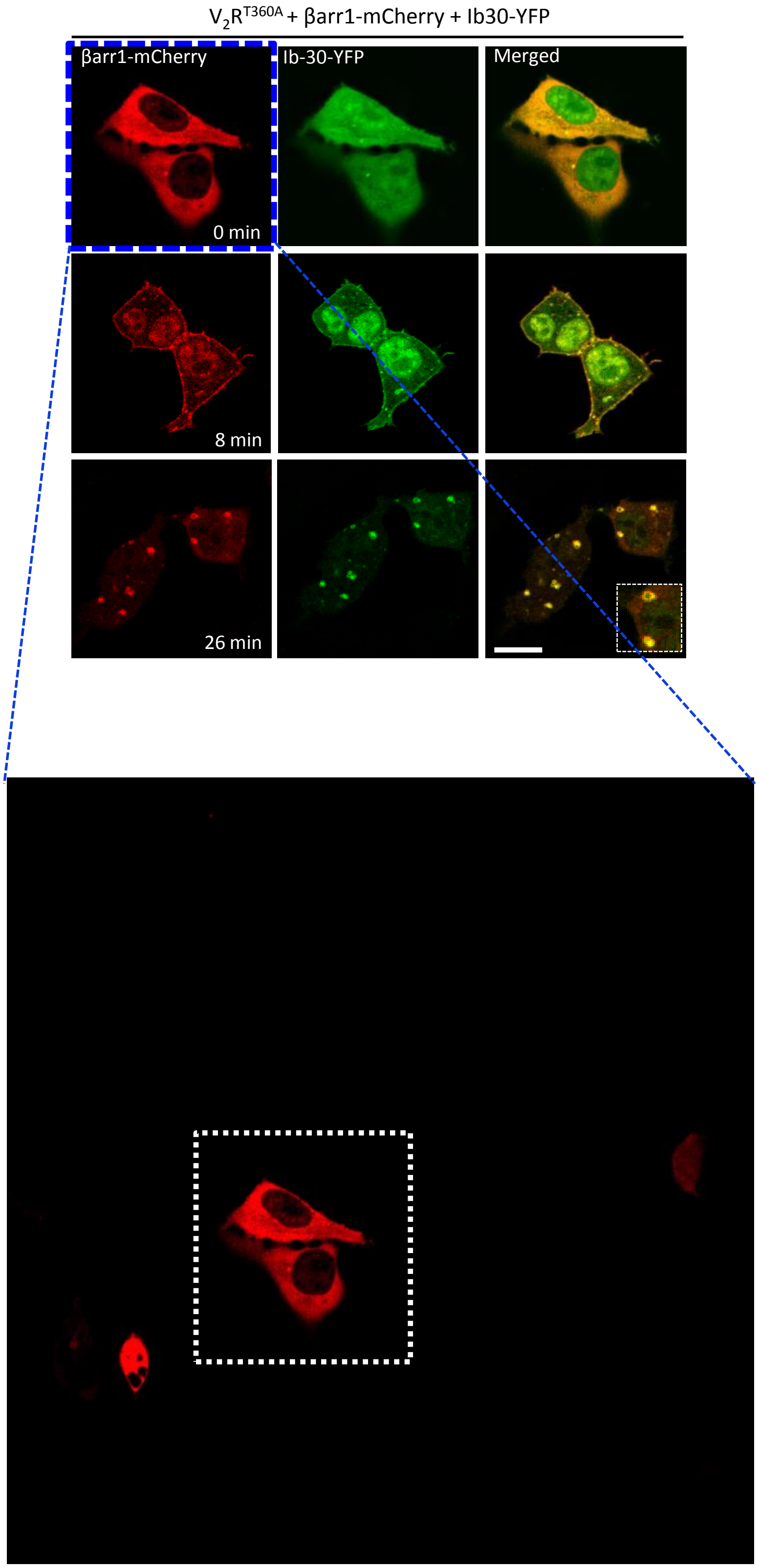

V_2R^{T360A} + βarr1-mCherry + Ib30-YFP
βarr1-mCherry
Ib-30-YFP
Merged
0 min
8 min
26 min

Figure 5c

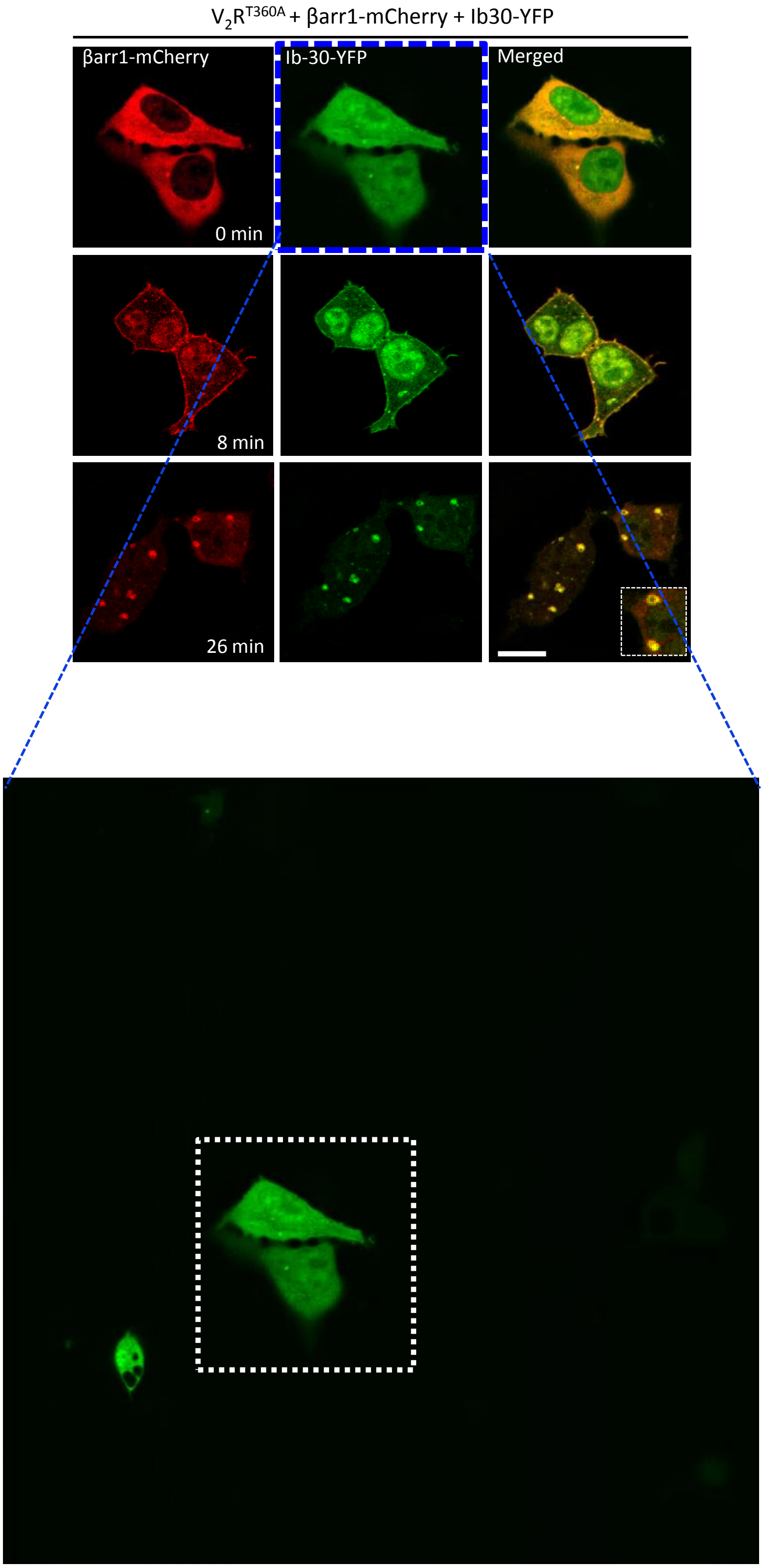

V2R T360A + βarr1-mCherry + Ib30-YFP
βarr1-mCherry
Ib-30-YFP
Merged
0 min
8 min
26 min

Figure 5c

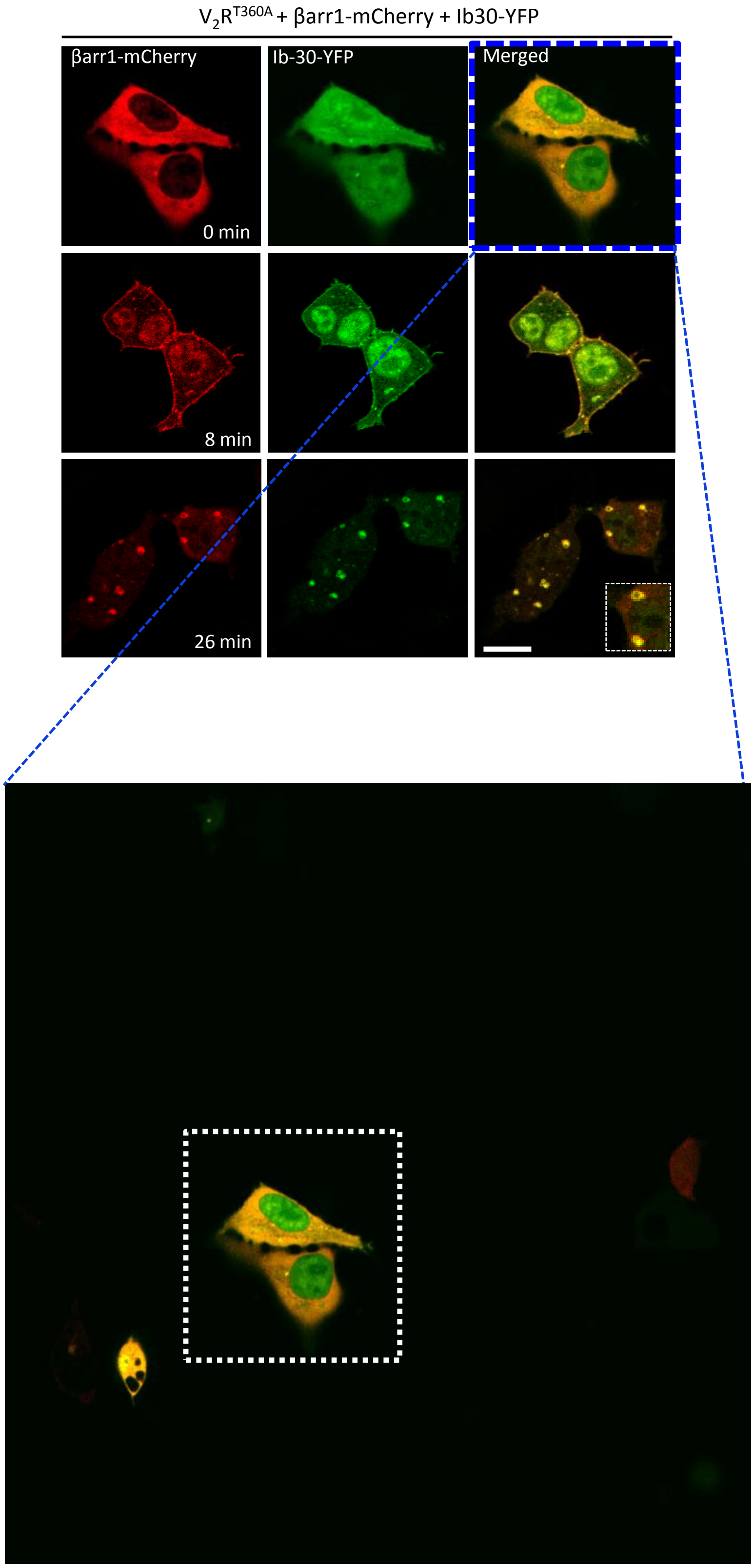
V$_2$R^{T360A} + βarr1-mCherry + Ib30-YFP
βarr1-mCherry
Ib-30-YFP
Merged
0 min
8 min
26 min

Figure 5c

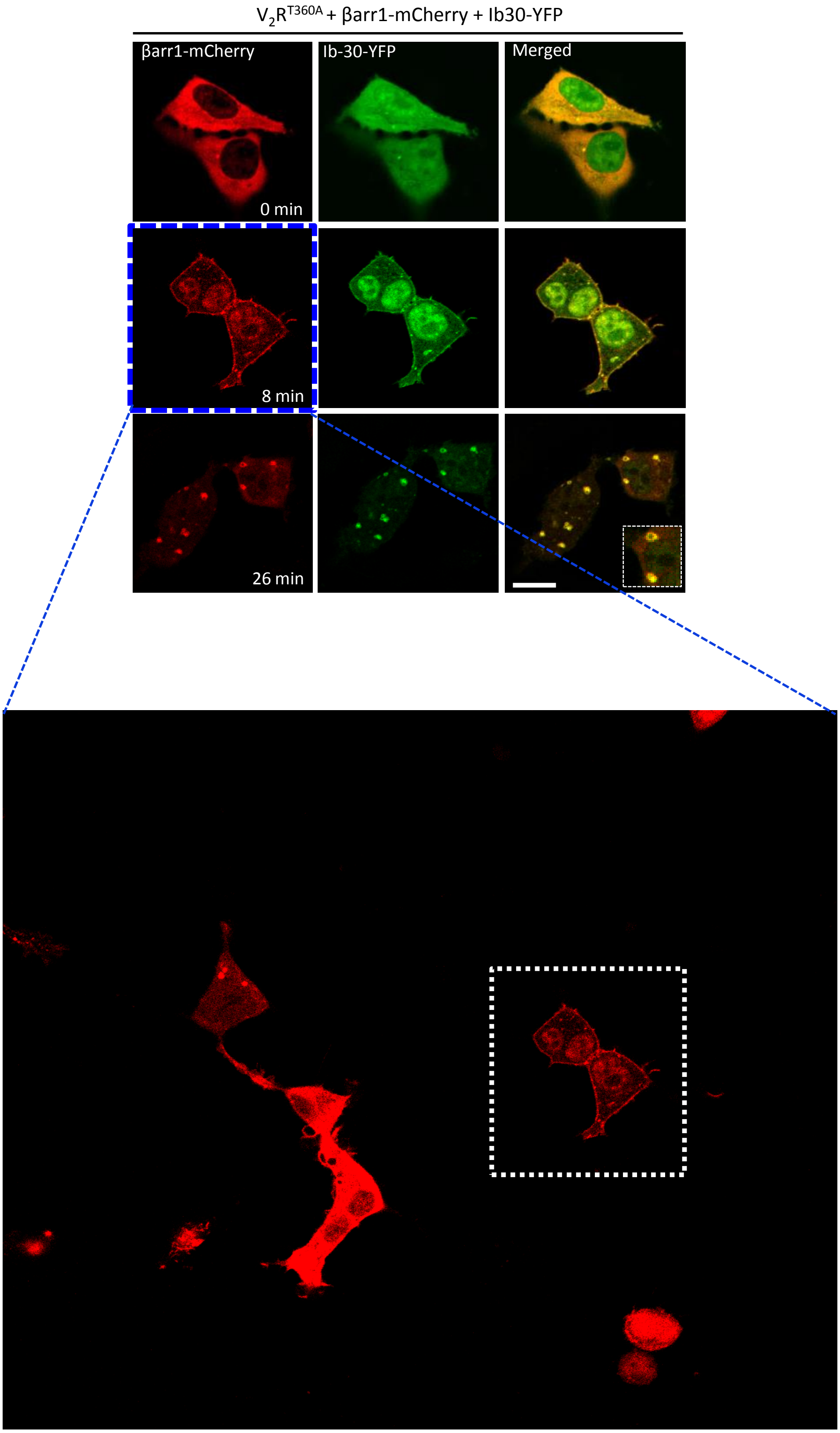

V2RT360A + βarr1-mCherry + Ib30-YFP
βarr1-mCherry
Ib-30-YFP
Merged
0 min
8 min
26 min

Figure 5c

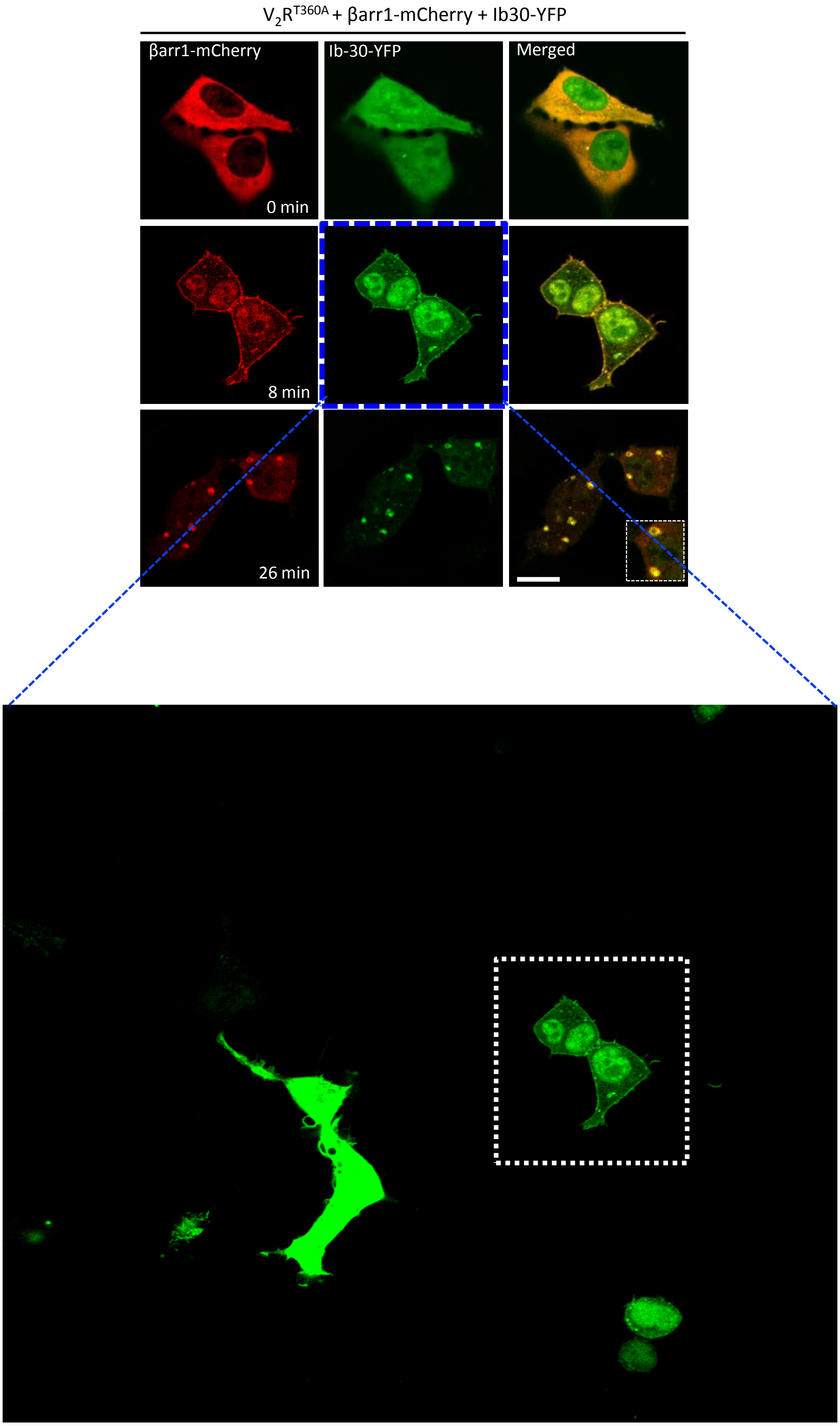

V_2R^{T360A} + βarr1-mCherry + Ib30-YFP
βarr1-mCherry
Ib-30-YFP
Merged
0 min
8 min
26 min

Figure 5c

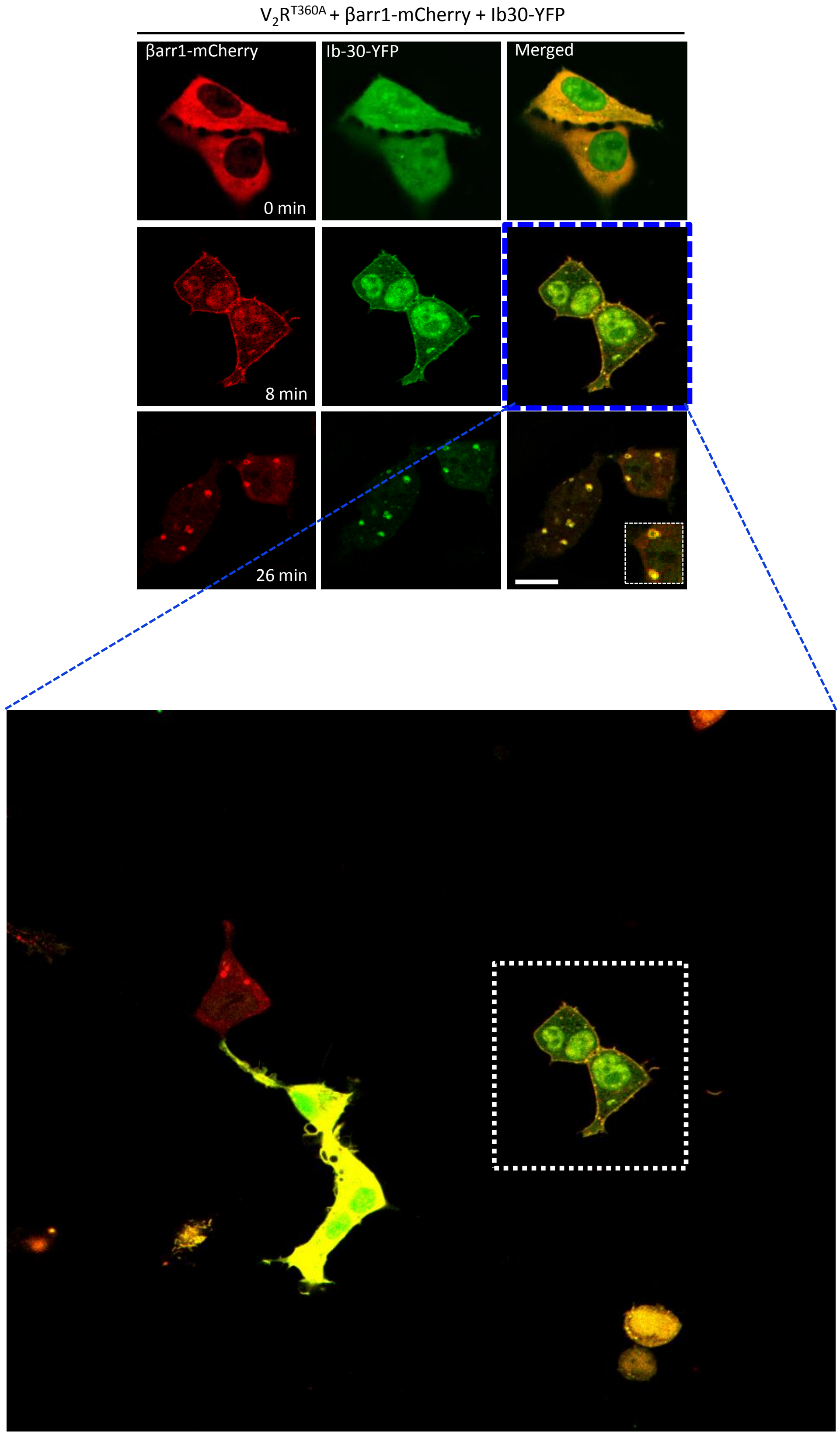

V_2R^{T360A} + βarr1-mCherry + Ib30-YFP
βarr1-mCherry
Ib-30-YFP
Merged
0 min
8 min
26 min

Figure 5c

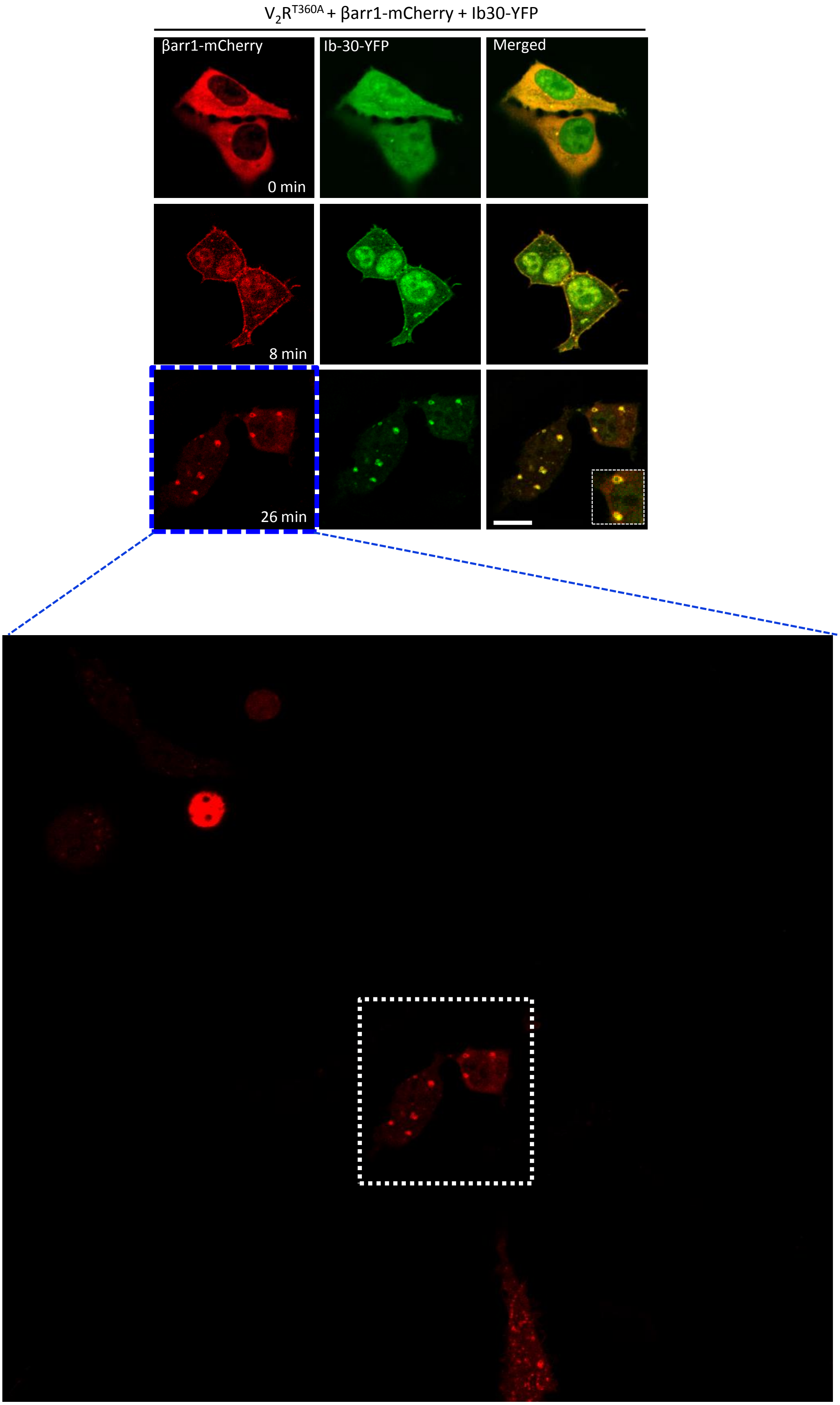

V2RT360A + βarr1-mCherry + Ib30-YFP
βarr1-mCherry
Ib-30-YFP
Merged
0 min
8 min
26 min

Figure 5c

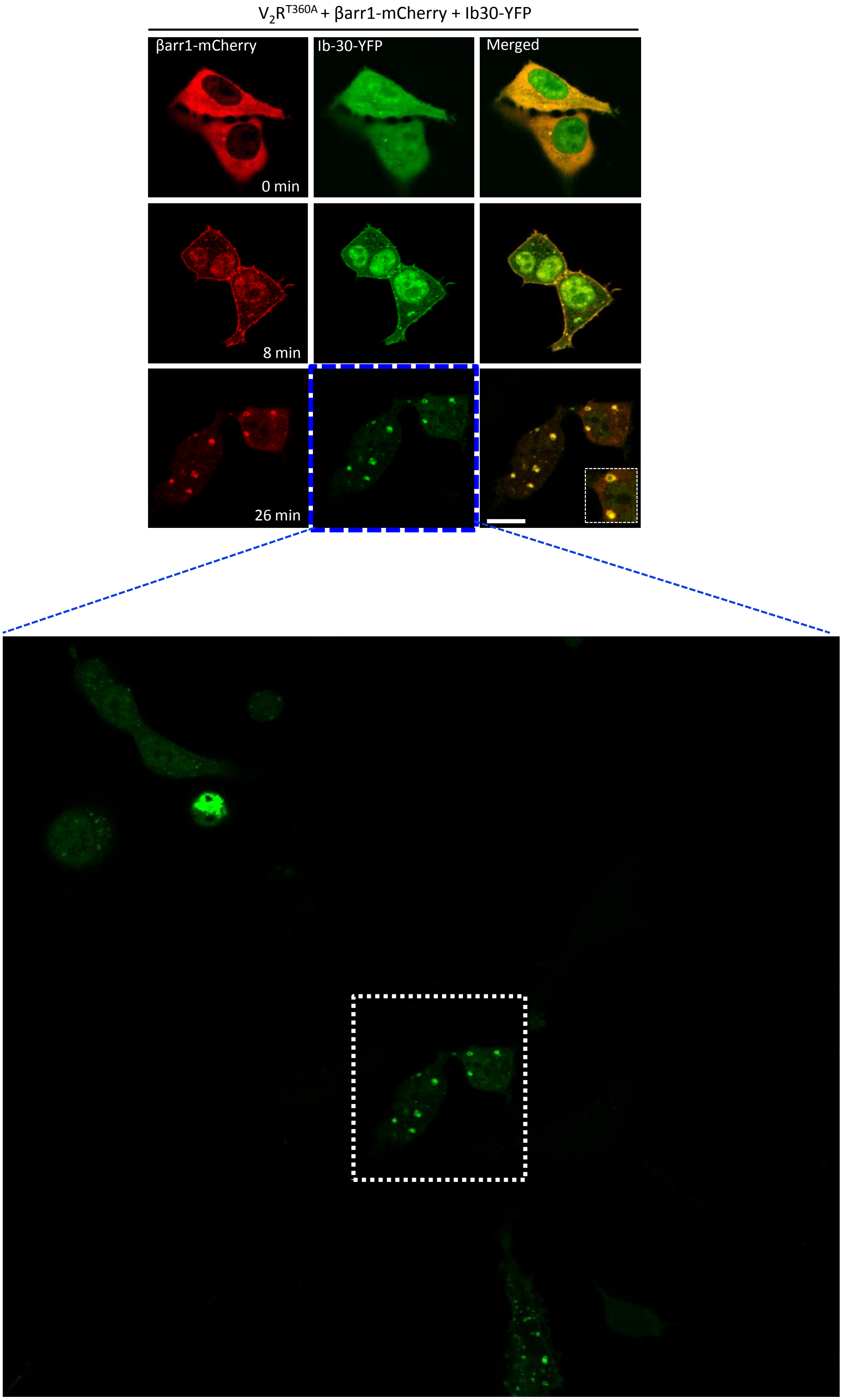

V2RT360A + βarr1-mCherry + Ib30-YFP
βarr1-mCherry
Ib-30-YFP
Merged
0 min
8 min
26 min

Figure 5c

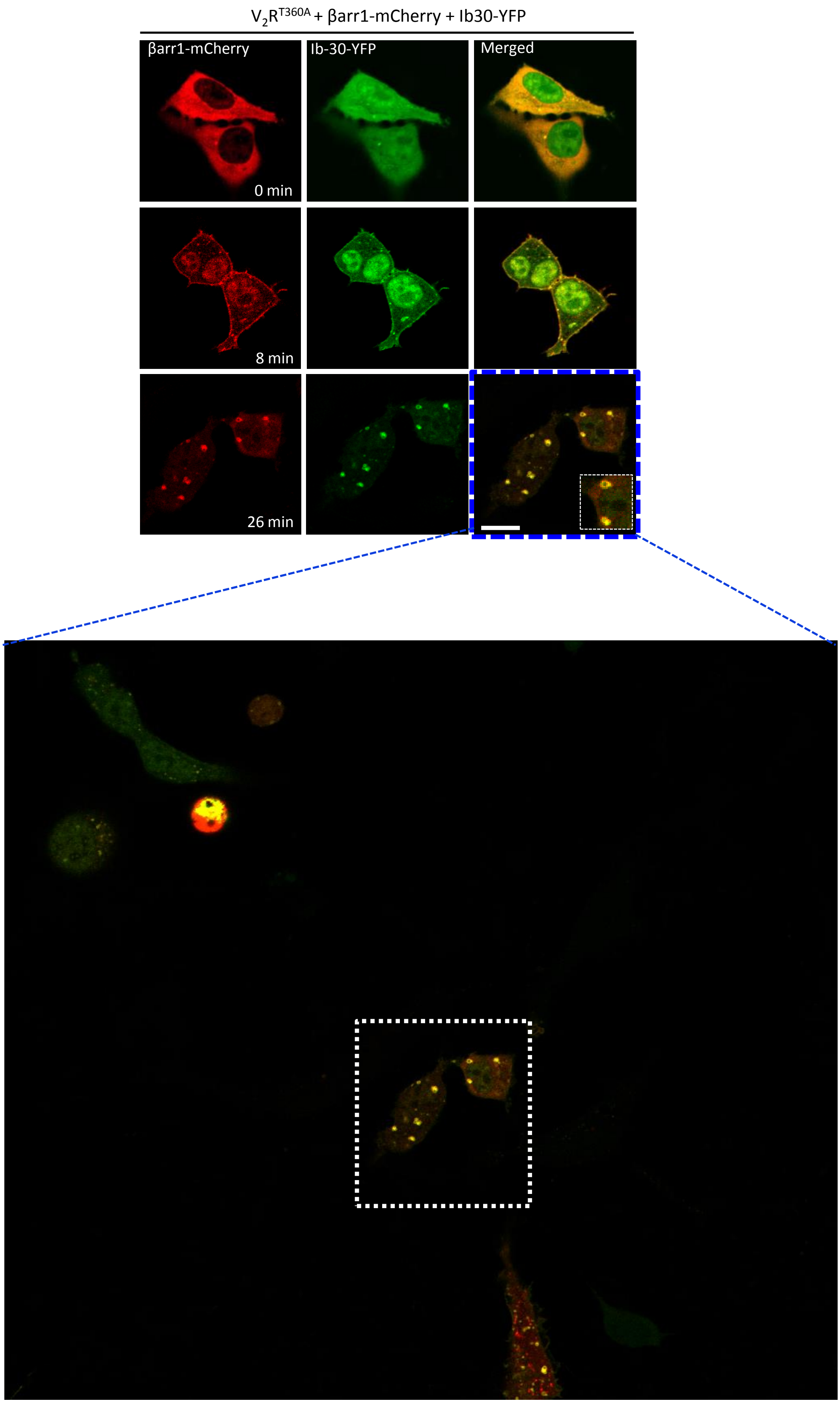

V_2R^{T360A} + βarr1-mCherry + Ib30-YFP
βarr1-mCherry
Ib-30-YFP
Merged
0 min
8 min
26 min

Figure 5e

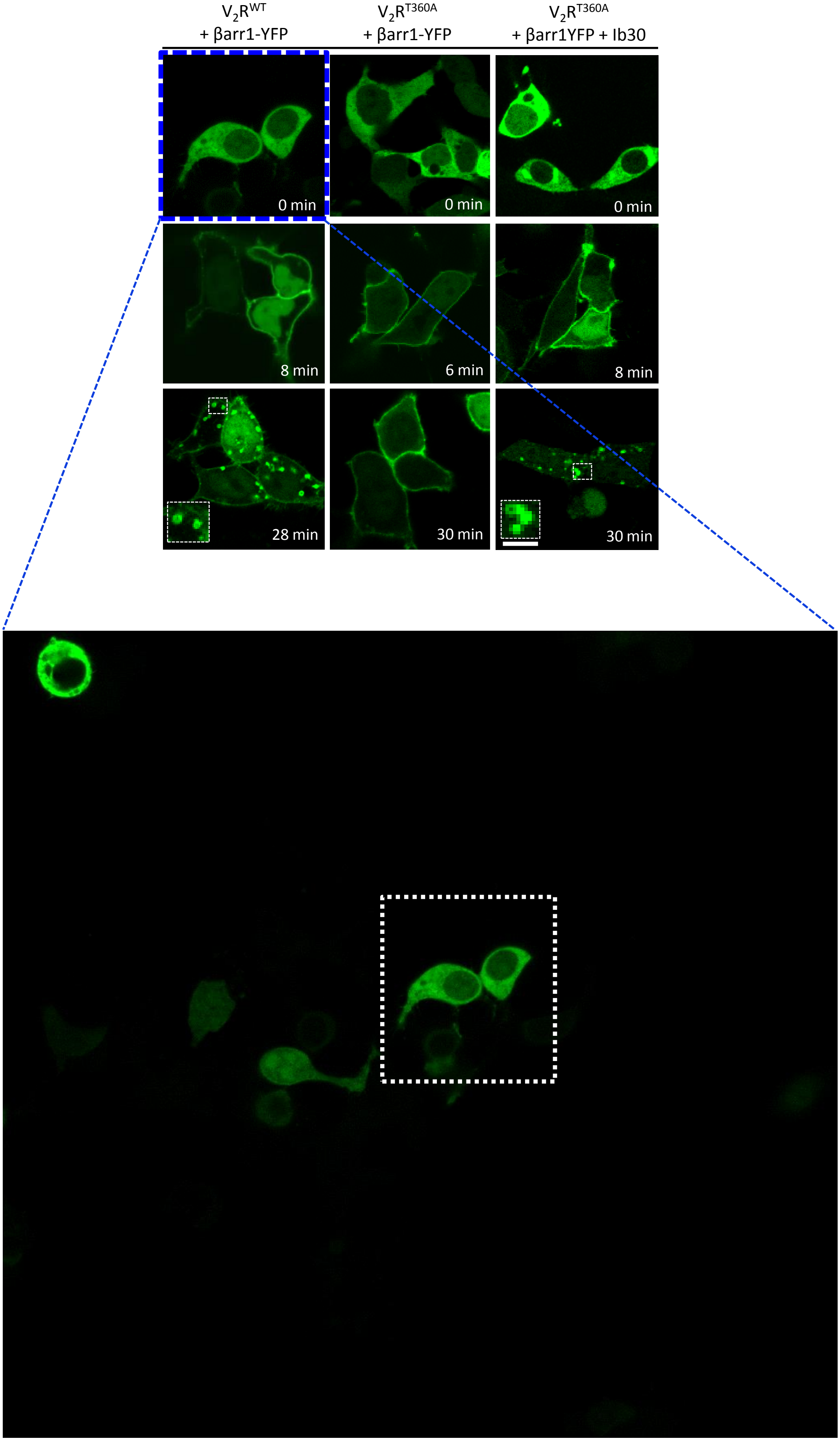
V_2R^{WT} + βarr1-YFP
V_2R^{T360A} + βarr1-YFP
V_2R^{T360A} + βarr1YFP + Ib30
0 min
0 min
0 min
8 min
6 min
8 min
28 min
30 min
30 min

Figure 5e

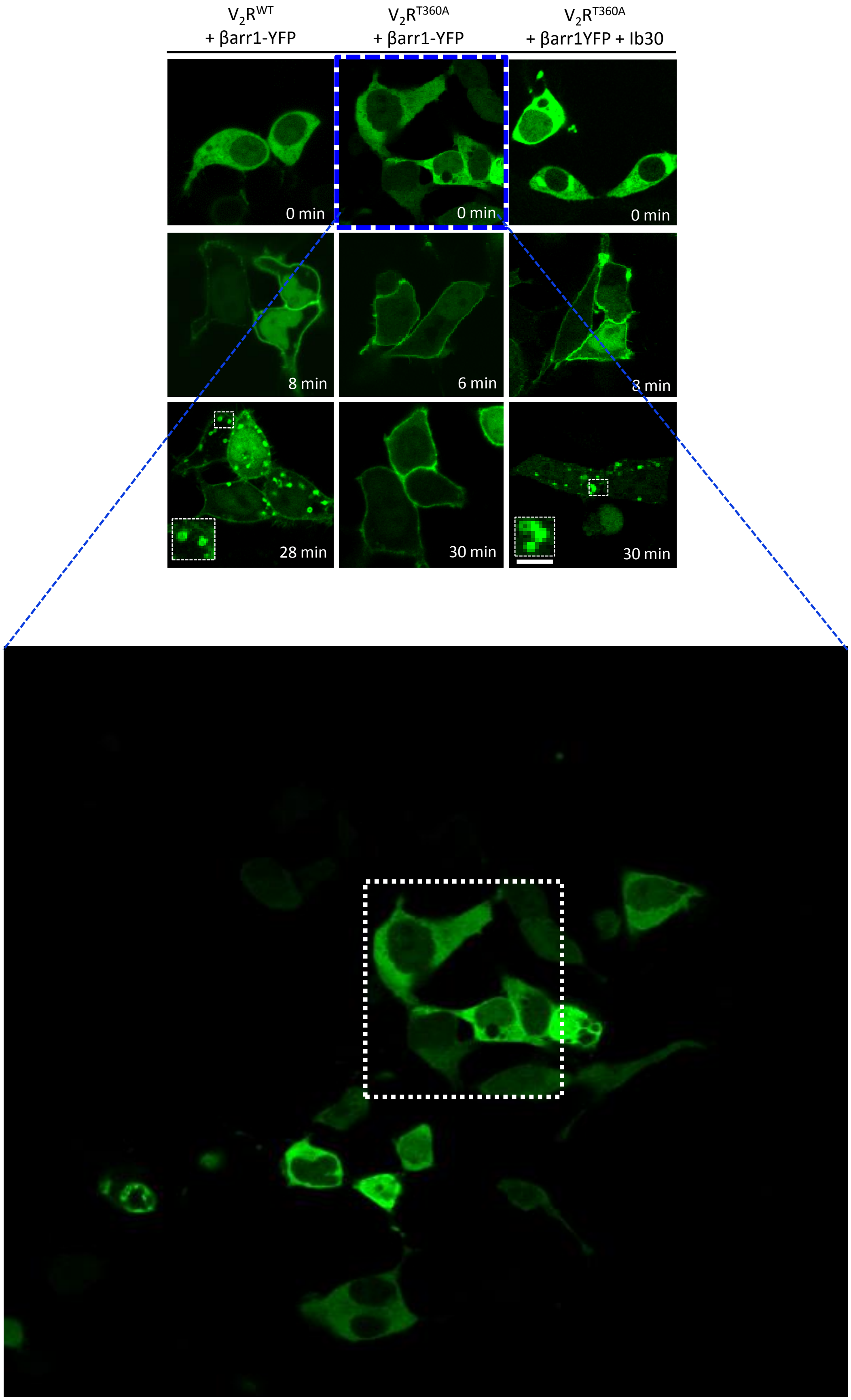

V2RWT + βarr1-YFP
V2RT360A + βarr1-YFP
V2RT360A + βarr1YFP + Ib30
0 min
0 min
0 min
8 min
6 min
8 min
28 min
30 min
30 min

Figure 5e

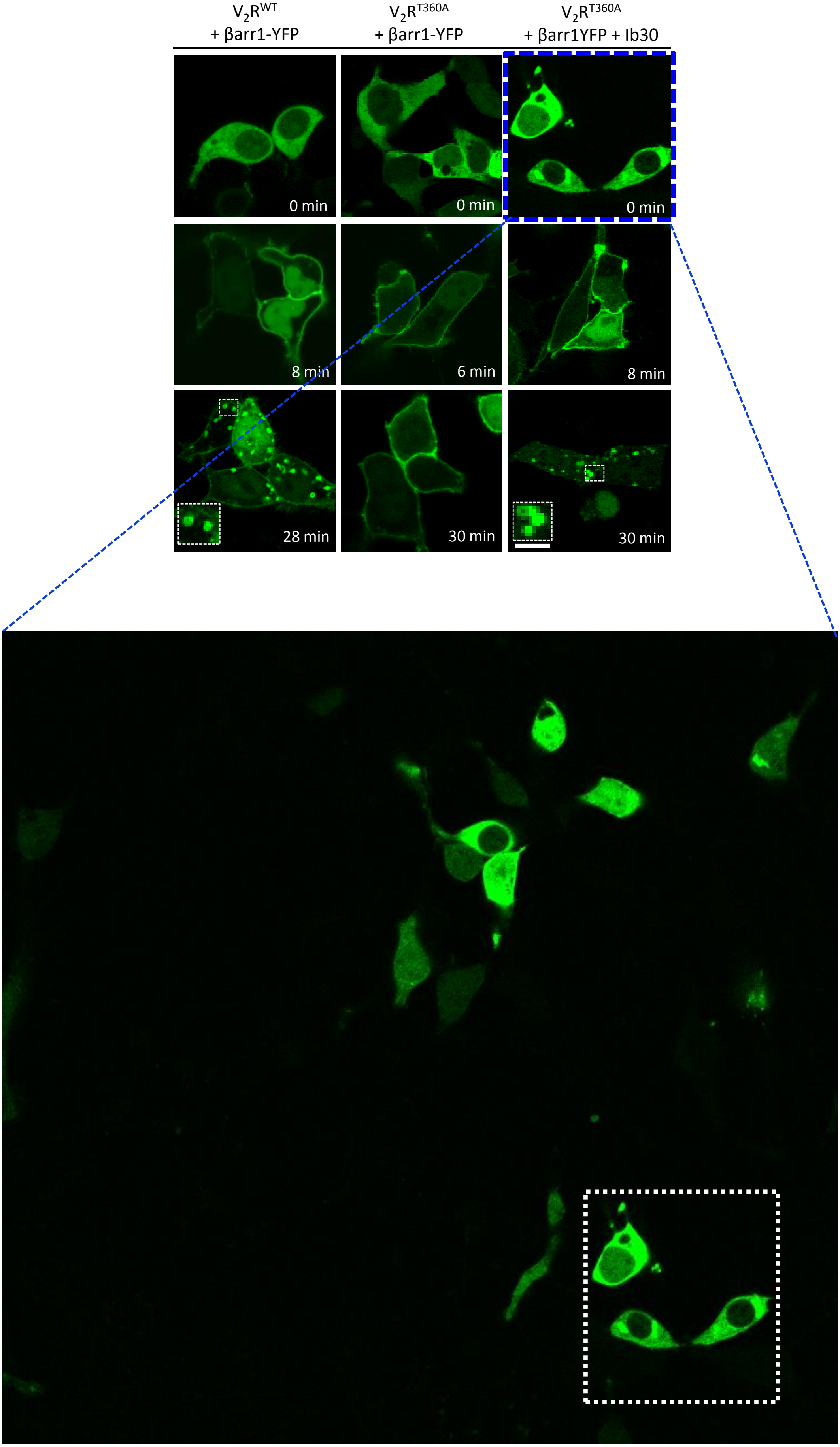
V_2R^{WT}
+ βarr1-YFP
V_2R^{T360A}
+ βarr1-YFP
V_2R^{T360A}
+ βarr1YFP + Ib30
0 min
0 min
0 min
8 min
6 min
8 min
28 min
30 min
30 min

Figure 5e

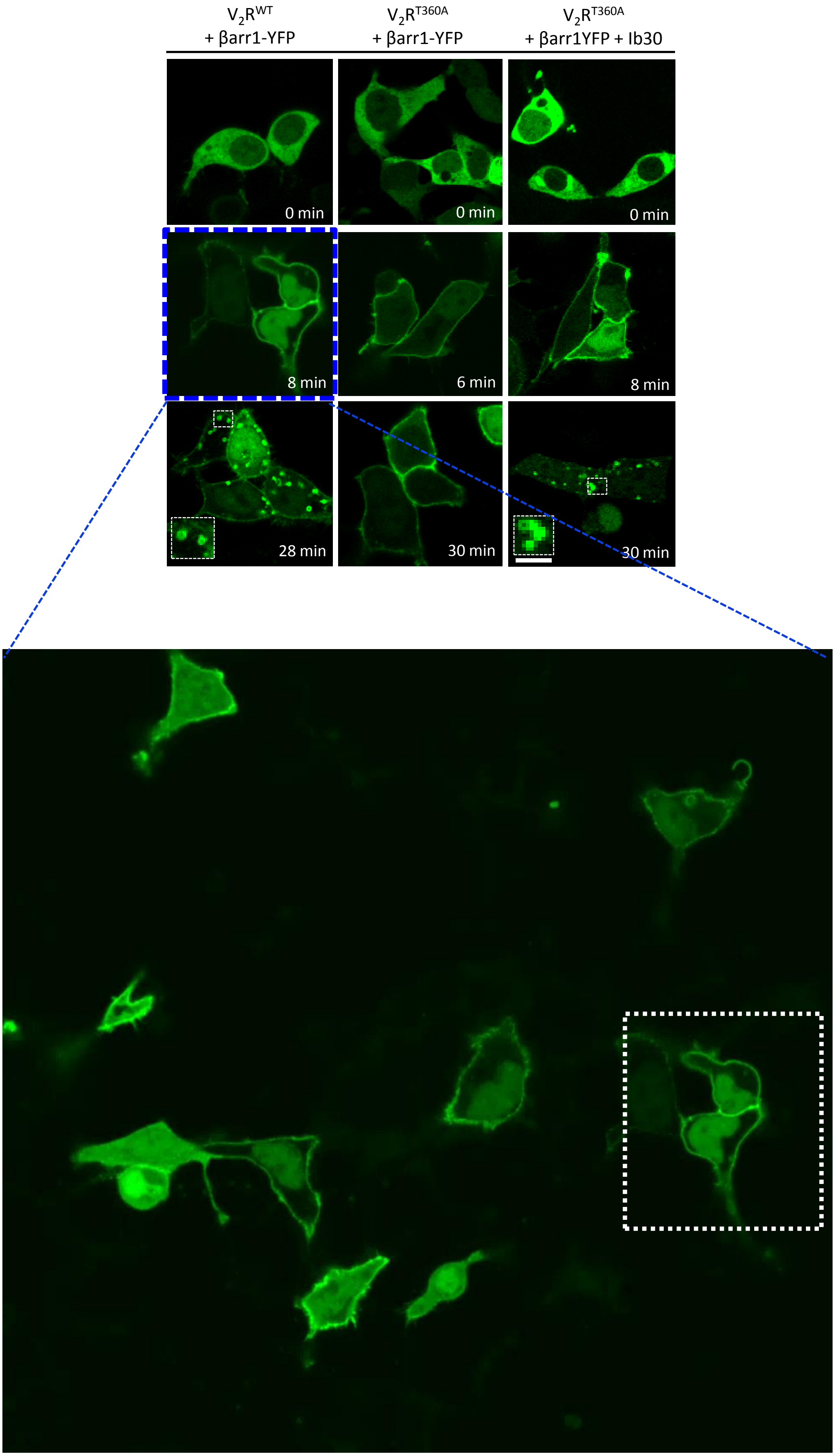

V_2R^{WT} + βarr1-YFP
V_2R^{T360A} + βarr1-YFP
V_2R^{T360A} + βarr1YFP + Ib30
0 min
0 min
0 min
8 min
6 min
8 min
28 min
30 min
30 min

Figure 5e

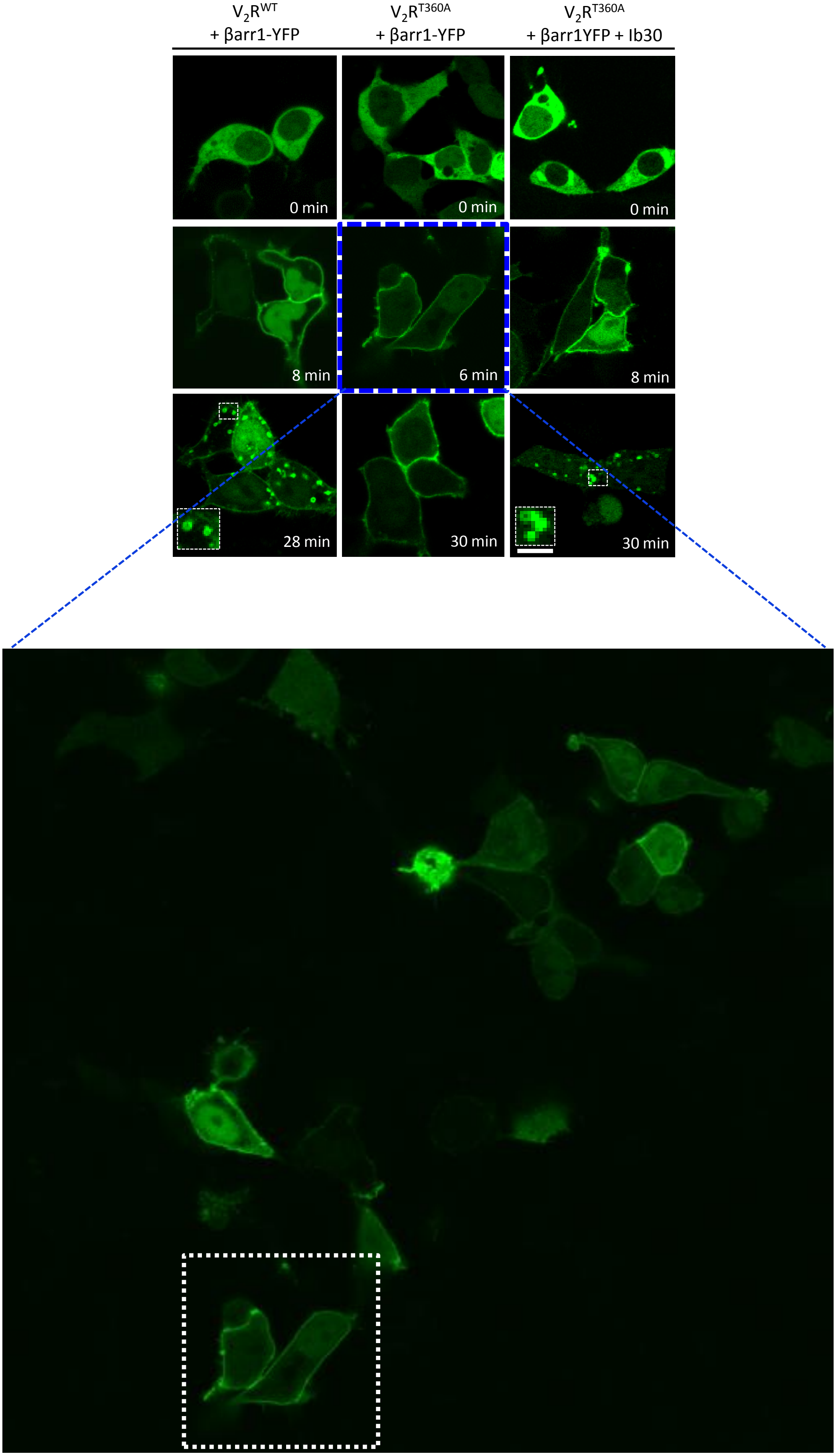
V_2R^{WT} + βarr1-YFP
V_2R^{T360A} + βarr1-YFP
V_2R^{T360A} + βarr1YFP + Ib30
0 min
0 min
0 min
8 min
6 min
8 min
28 min
30 min
30 min

Figure 5e

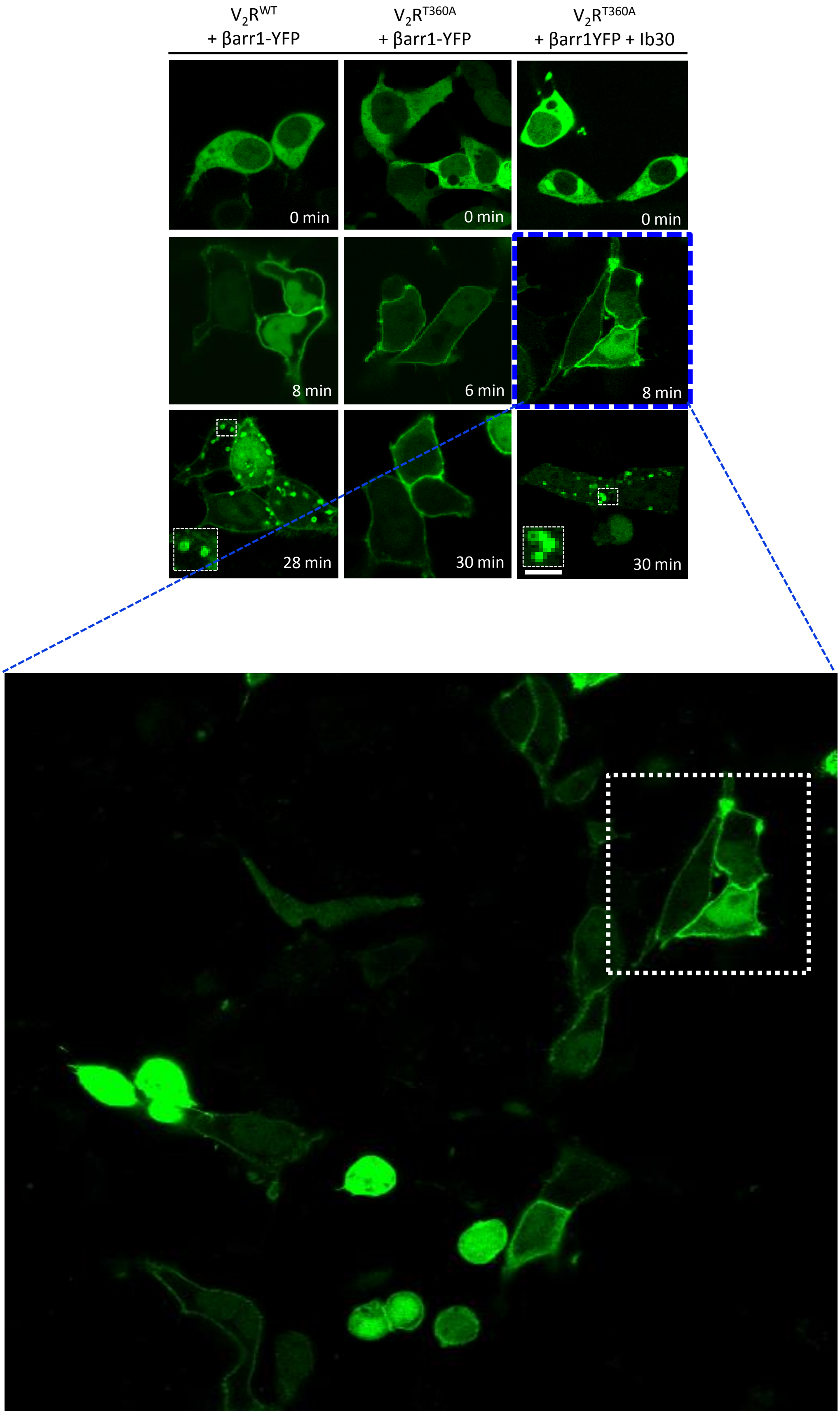
V_2R^{WT} + βarr1-YFP
V_2R^{T360A} + βarr1-YFP
V_2R^{T360A} + βarr1YFP + Ib30
0 min
0 min
0 min
8 min
6 min
8 min
28 min
30 min
30 min

Figure 5e

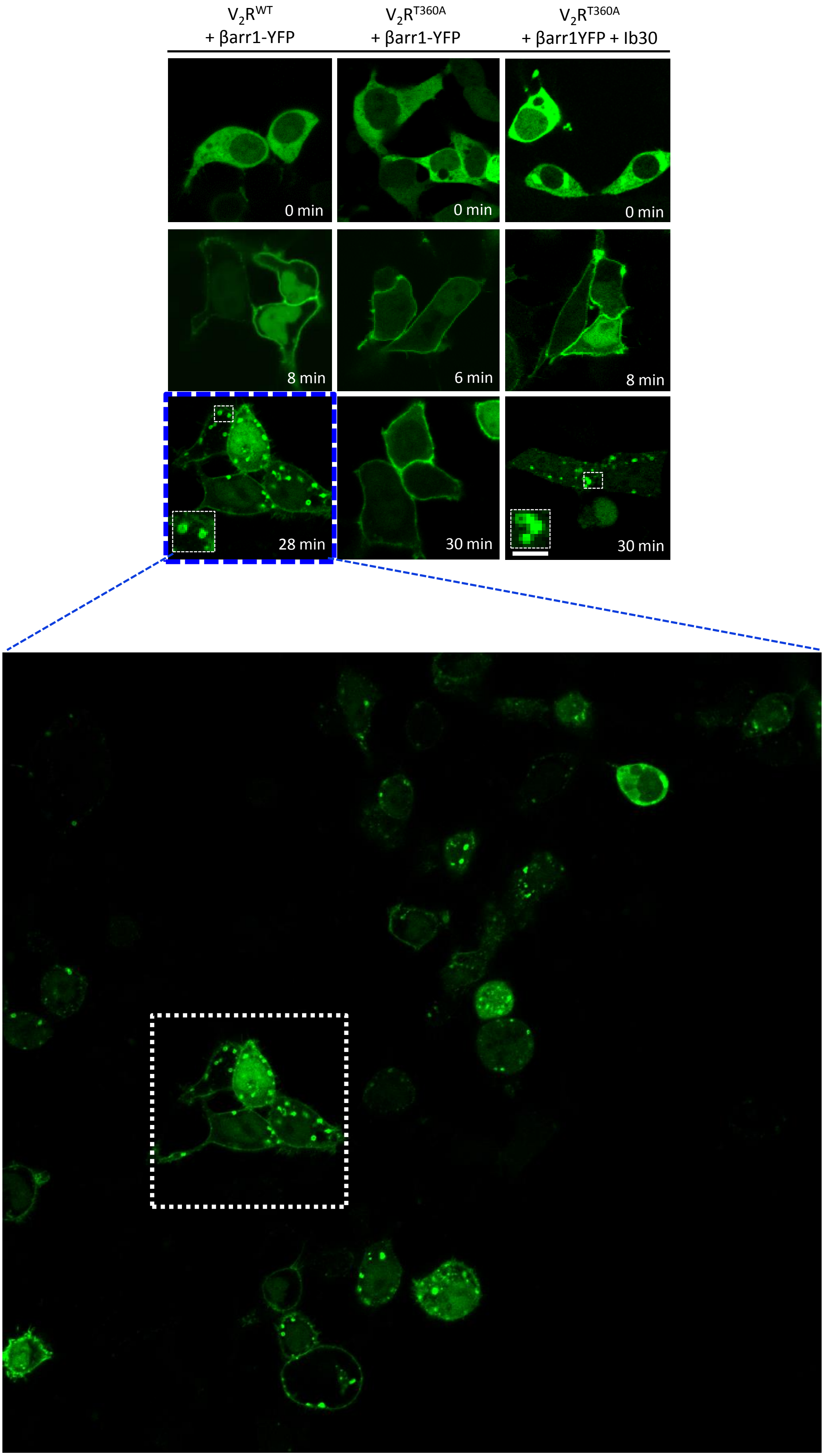

V_2R^{WT} + βarr1-YFP
V_2R^{T360A} + βarr1-YFP
V_2R^{T360A} + βarr1YFP + Ib30
0 min
0 min
0 min
8 min
6 min
8 min
28 min
30 min
30 min

Figure 5e

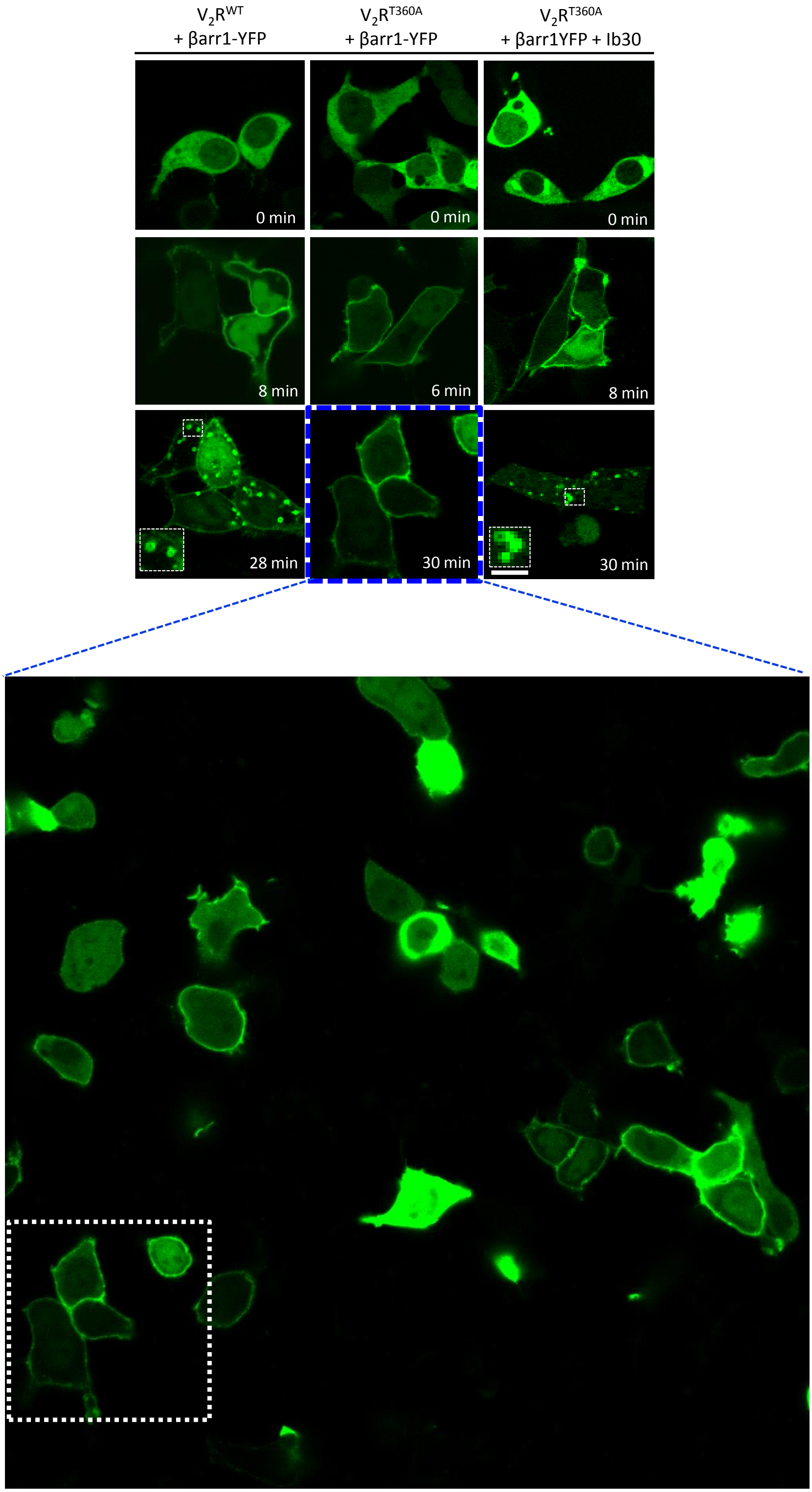

V_2R^{WT}
+ βarr1-YFP
V_2R^{T360A}
+ βarr1-YFP
V_2R^{T360A}
+ βarr1YFP + Ib30
0 min
0 min
0 min
8 min
6 min
8 min
28 min
30 min
30 min

Figure 5e

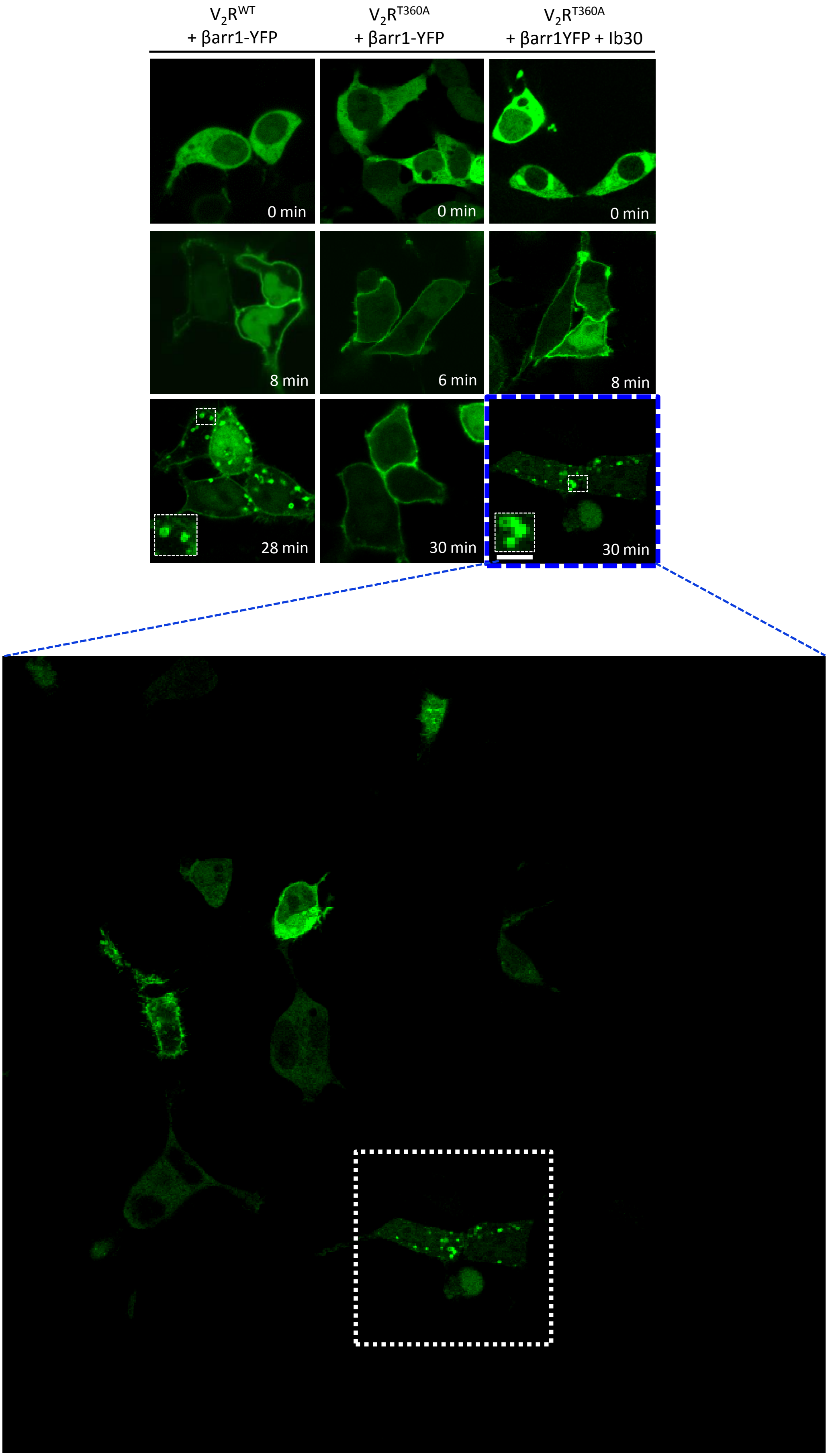

V_2R^{WT} + βarr1-YFP
V_2R^{T360A} + βarr1-YFP
V_2R^{T360A} + βarr1YFP + Ib30
0 min
0 min
0 min
8 min
6 min
8 min
28 min
30 min
30 min

Figure 7a

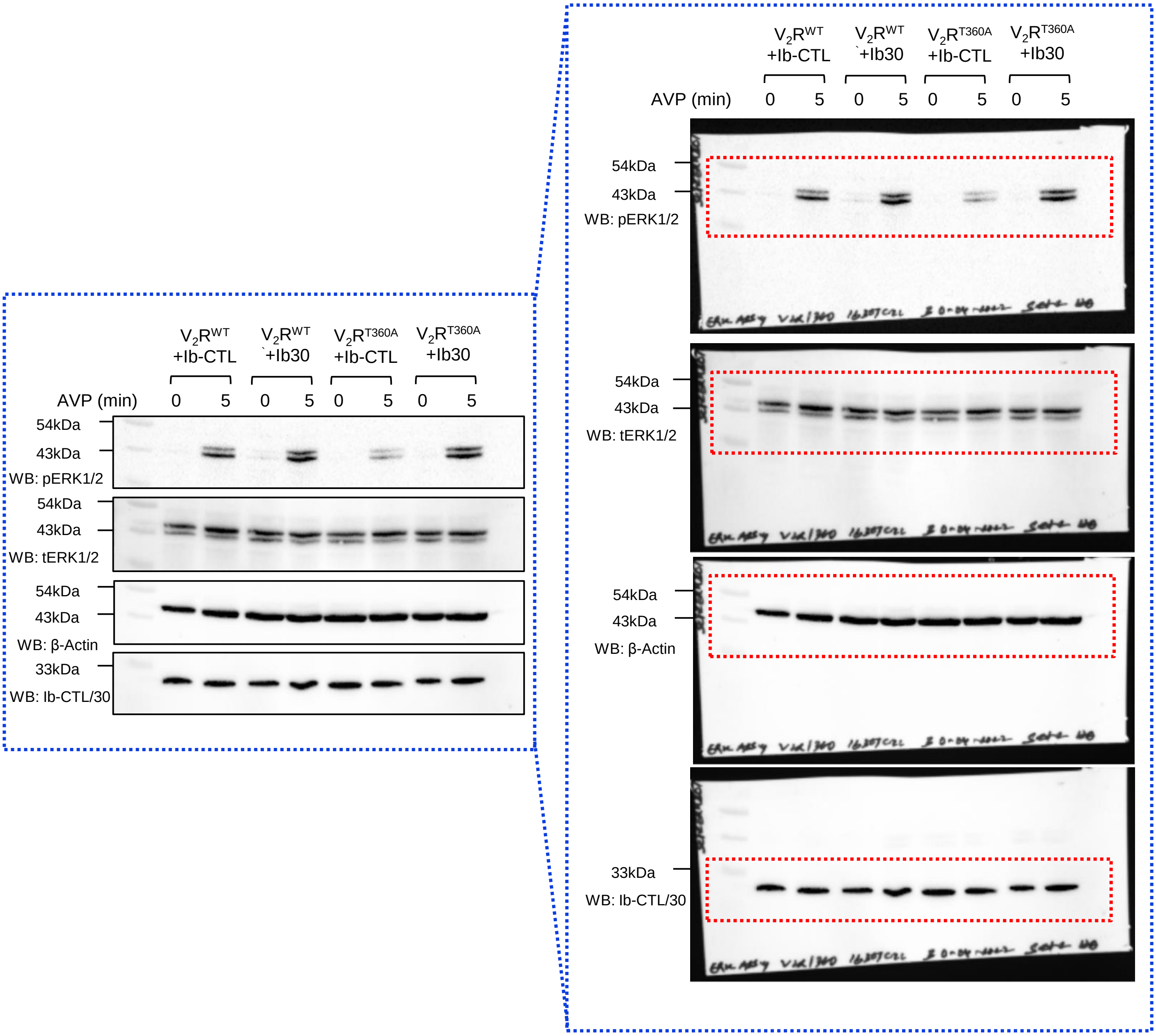
V2RWT +Ib-CTL
V2RWT `+Ib30
V2RT360A +Ib-CTL
V2RT360A +Ib30
AVP (min) 0 5 0 5 0 5 0 5
54kDa
43kDa
WB: pERK1/2
54kDa
43kDa
WB: tERK1/2
54kDa
43kDa
WB: β-Actin
33kDa
WB: Ib-CTL/30

Figure 9a

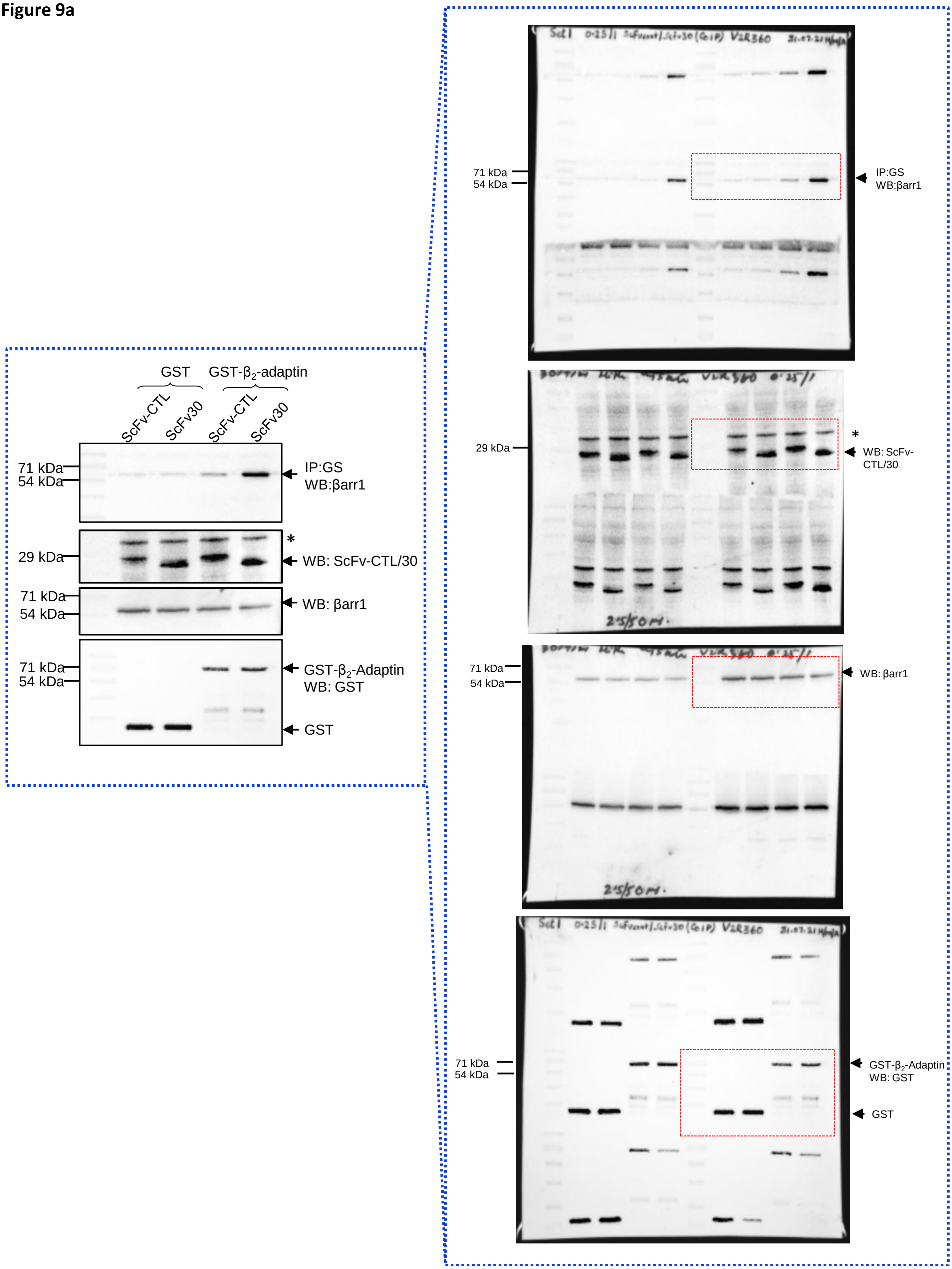
GST
GST-β2-adaptin
ScFv-CTL
ScFv30
ScFv-CTL
ScFv30
71 kDa
54 kDa
IP:GS
WB:βarr1
*
29 kDa
WB: ScFv-CTL/30
71 kDa
54 kDa
WB: βarr1
71 kDa
54 kDa
GST-β2-Adaptin
WB: GST
GST
71 kDa
54 kDa
IP:GS
WB:βarr1
29 kDa
*
WB: ScFv-CTL/30
71 kDa
54 kDa
WB: βarr1
71 kDa
54 kDa
GST-β2-Adaptin
WB: GST
GST

Figure 9e

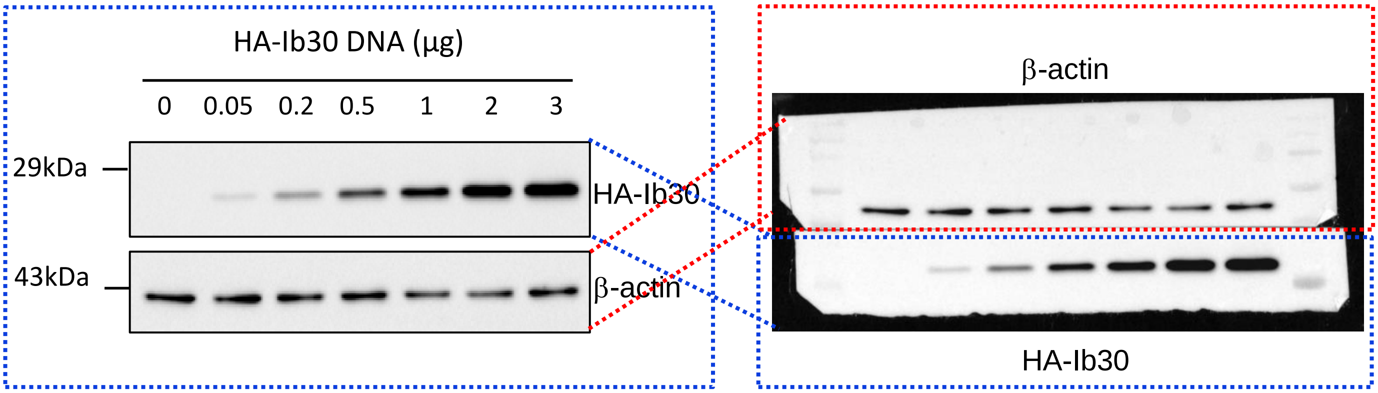
HA-Ib30 DNA (µg)
0
0.05
0.2
0.5
1
2
3
29kDa
43kDa
HA-Ib30
β-actin
β-actin
HA-Ib30

Supplementary Figure 1a

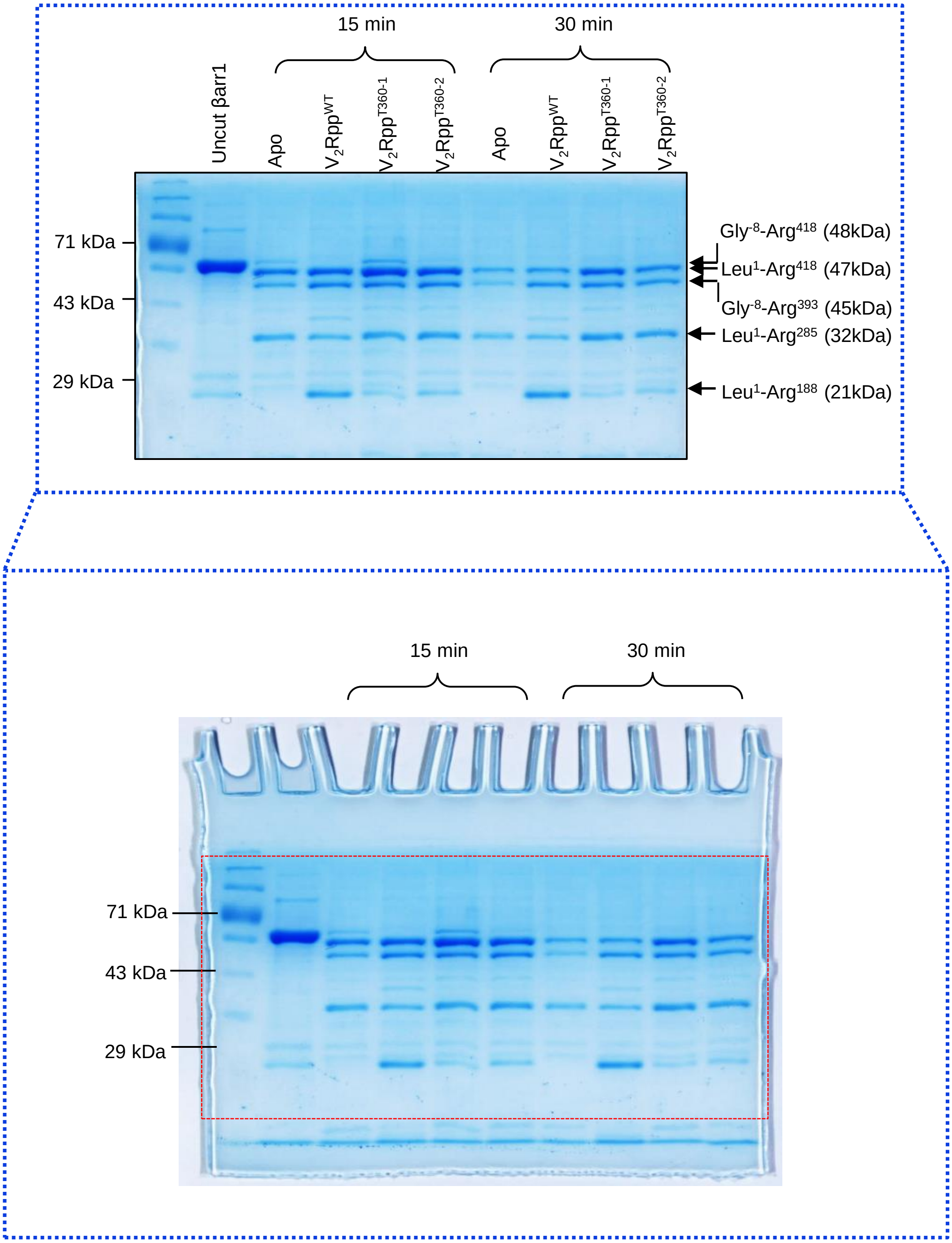

Supplement: Supplementary file 4 — Source Data [file 41467_2022_32386_MOESM4_ESM.zip › Source data-uncropped blot images and micrographs.pdf]
